# Structure of defense against restriction proteins DarA and Hdf in phage P1 reveals a new molecular mechanism during phage assembly, infection and DNA ejection

**DOI:** 10.1371/journal.ppat.1013869

**Published:** 2026-01-16

**Authors:** Jing Zheng, Yuan Chen, Siting Chen, Junquan Zhou, Hao Xiao, Fan Yang, Hongrong Liu

**Affiliations:** 1 Institute of Interdisciplinary Studies, Key Laboratory for Matter Microstructure and Function of Hunan Province, Key Laboratory of Low-dimensional Quantum Structures and Quantum Control, School of Physics and Electronics, Hunan Normal University, Changsha, China; 2 Hunan Research Center of the Basic Discipline for Quantum Effects and Quantum Technologies, Hunan Normal University, Changsha, China; Tufts University School of Medicine, UNITED STATES OF AMERICA

## Abstract

The continuous “arms race” between bacterial antiviral defense systems and phage anti-defense strategies drives evolutionary innovation. Previous study indicated that the defense against restriction (Dar) proteins DarA and Hdf in myophage P1 are associated with the head morphogenesis. However, the structural information for these proteins was lacking, and the mechanisms by which they mediate head morphogenesis and protect phage DNA against bacterial defense systems remained poorly understood. Using cryo-electron microscopy (cryo-EM), we resolved the entire structures of extended P1 and contracted P1 with partial DNA, with the latter lacking the baseplate, as well as the head structure of contracted P1 without DNA. We identified the structural proteins for the P1, including the head, connector complex, and baseplate, which exhibited conserved properties among the majority of myophages with a simple baseplate. Notably, 55 DarA-Hdf pairs are attached to the inner surface of head at each penton-hexon junction in the extended P1 and contracted P1 with partial DNA. The DarA and Hdf together form a complex that is tightly bound to the capsid and interacts with the DNA. However, these pairs are absent in the contracted P1 without DNA. Based on our three states of P1, we hypothesis that these extensive interactions among DarA, Hdf, DNA, and head play crucial roles in mediating capsid assembly, enhancing capsid stability, and protecting phage DNA. Our results provide a structural basis for further exploration of the mechanism by which Dar proteins function during phage assembly, infection and DNA ejection. This molecular mechanism may be conserved among P1-like phages.

## Author summary

Bacteria have evolved a range of defense mechanisms, such as restriction-modification (R-M) systems, to destroy invading phage DNA, posing a major challenge to phage therapy. However, phages have evolved counter-defenses. Notably, myophage P1 encodes defense against restriction (Dar) proteins, including DarA and Hdf, to protect its genome. However, the structural basis and protection mechanism for these Dar proteins remain to be elucidated. In this study, we focused on the head structures of the bacteriophage P1 in its extended state and two types of contracted state. We discovered that DarA and Hdf proteins form 55 pair complexes that are anchored at each penton-hexon junction on the inner surface of the head in the extended P1 and the contracted P1 with partial DNA. The complex has a positively charged inner surface, thus interacting with the DNA. Additionally, the complex has a hydrophilic outer surface that maybe necessary to maintain the solubility of the DNA-protein complex in the host cytoplasm following injection. However, these 55 pairs are absent in the contracted P1 without DNA. This study unveils the molecular details of intricate interactions among DarA, Hdf, DNA, and capsid in different states, thereby uncovering a sophisticated phage defense mechanism and providing structural insights for engineering phages to better overcome bacterial resistance.

## Introduction

Bacteria and their viral predators, bacteriophages (phages), are engaged in a perpetual battle [1]. In order to defend against the phage infection, bacteria have evolved a range of defense systems, including abortive infection [2], restriction-modification (R-M) systems [3], resistant variants [4], and CRISPR-Cas systems [5,6]. Among these, the R-M system is the most common defense mechanism, as we know so far, being encoded in approximately 90% of bacterial genomes [7]. This system is characterized by its ability to recognize and cleave phage DNA in a site-specific manner, thereby protecting self-DNA. In response, phages have evolved diverse counter-strategies to evade bacterial defense [8,9], such as encoding protein inhibitors that inactivate defense systems [10–12], hijacking host antitoxins to prevent abortive infection [13,14] and employing anti-CRISPR proteins to evade the CRISPR–Cas [15,16]. The comprehension of phage anti-defense mechanisms holds considerable potential for applications in antibacterial therapy, gene editing tools, and biotechnology [1].

R-M systems can be classified into four major types (I-IV) based on their DNA cleavage mechanisms, subunit compositions, and DNA recognition sequences [17,18]. Genomic sequencing analysis indicates that the type I R-M systems are particularly prevalent, existing in approximately 50% of bacteria and archaea, and demonstrating their significance in the phage defense systems [18]. Some phages, such as T4 [19–21] and P1 [22–24], encode internal proteins packaged within their head that are co-injected with DNA into the host during infection. These proteins are pivotal in defensing against different types of R-M systems. However, the structural details of these internal proteins within the head and the precise mechanism of co-release with DNA remained to be elucidated.

P1 is an important *Escherichia coli* phage with clinical significance [25]. Owing to its broad host range [24], transduction capability [26], and lysogenic properties [27], P1 has played an important role in the study of fundamental molecular processes, the genetic mapping of *E. coli* chromosomes, and the development of genetic engineering technologies [28–30]. For instance, the P1 phagemid system facilitates the efficient delivery of the chromosomal-targeting cas9 constructs into both *E. coli* and the dysentery-causing pathogen *Shigella flexneri*, resulting in sequence-specific bacterial lethality [30]. A key feature of P1 is its Dar system, a representative defense against type I R-M systems, composed of at least six internal proteins: DarA, DarB, DdrA, DdrB, Hdf and Ulx [22,24]. Biochemical studies demonstrated that P1 Dar proteins are co-ejected with phage DNA into the host during the initiation of infection, subsequently protecting the DNA from restriction by different type I R-M subsystems [23]. DarA is required for protection against EcoA and StySA type I R-M systems, while Ulx, DarB, and DdrB have been shown to confer sensitivity to EcoB and EcoK type I R-M systems [22,23]. Importantly, DarA and Hdf play a role not only in the anti-defense system but also in head morphogenesis [22]. Nevertheless, their three-dimensional (3D) structures, precise locations within the head, and the molecular mechanisms mediating head morphogenesis and co-releasing with DNA remained poorly understood. The deletion of either DarA or Hdf results in P1 particles lacking all other Dar proteins [22], suggesting the paired appearance of Hdf and DarA, and an interdependence between these proteins for phage assembly. Notably, P1-like phages constitute a large and diverse group. Genome sequencing has revealed that P1-like phages, such as *Salmonella* phage SJ46 [31], *E. coli* phages RCS47 [32], and JL22 [33], encode DarA and Hdf homologs. However, the structure and function of these homologs had not been reported.

P1 exhibits a typical myophage morphology, possessing an icosahedral head, a long and contractile tail, and a baseplate [34]. The tail is attached to a unique 5-fold vertex of the head via a connector complex. In our preceding report, the structures of tail terminator, tube, and sheath proteins of P1 in the extended and contracted states were presented, thereby demonstrating that the tail contraction propagates in a wave-like manner [34]. However, the structural information of other proteins was lacking, hindering a comprehensive understanding of P1 function. In this paper, using cryo-EM, we determined the structures of the P1 in extended state, and two types of contracted states. The density maps enabled us to identify almost all proteins from the head to the tail, including the head (gp23, DarA and Hdf), the connector complex (Prt, PmgC, and gp7), and the baseplate (Tub, PmgG, gp6, gp5, UpfC, UpfB, PmgA, gp16, Bp1A and gp26). Significantly, 55 DarA-Hdf pairs are located at each junction of penton and hexon on the capsid’s inner surface, forming close interactions with both the capsid and DNA in the extended P1 and contracted P1 with partial DNA. However, these pairs are absent in the contracted P1 without DNA. These interactions among DarA, Hdf, DNA, and capsid provide a structural basis for head assembly, co-release of the DNA and Dar proteins, and the defense against the R-M system—a mechanism potentially conserved among P1-like phages.

## Results

### Overall structures of myophage P1 in extended and contracted states

**Fig 1:**
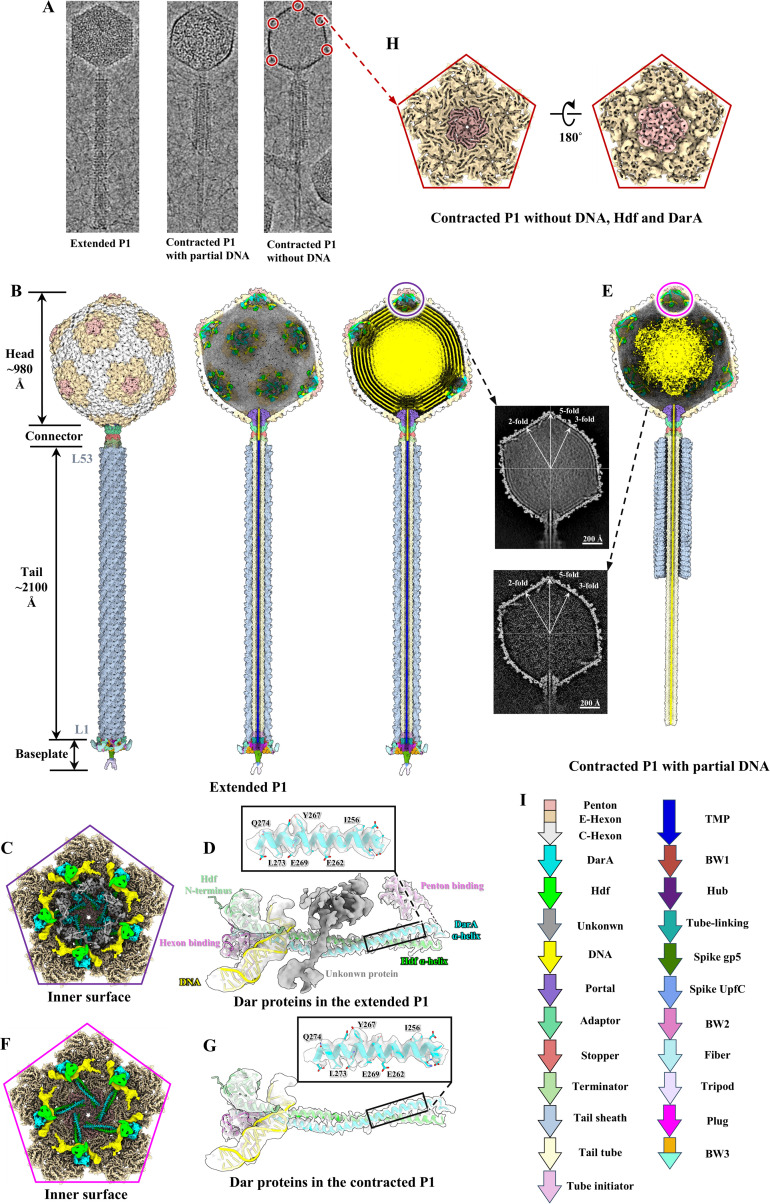
Structures of myophage P1 with three states, including the extended P1, the contracted P1 with partial DNA and the contracted P1 without DNA. **(A)** Representative cryo-EM images of the three states of P1. The icosahedral vertices of the contracted P1 without DNA were boxed. **(B)** Side (left), cut-open with manually removed DNA (middle) and cut-open (right) views of the intact structure of extended P1. L1 and L53 denote the first and final layers of the tail sheath, respectively. The inset shows the central cross-section of the head-connector in the extended P1. **(C)** Zoomed-in view of the inner surface around an icosahedral vertex to show the interactions among DarA, Hdf, DNA, a mass of low-resolution density (the unkonwn protein) and capsid in the extended P1. **(D)** Zoomed-in view of density maps (transparency) of Dar proteins and DNA in the extended P1 superimposed on its atomic models. The atomic models of the α-helix domain of DarA (cyan) and Hdf (lime) were manually built. The atomic models of the hexon and penton binding domains (magenta) of DarA were obtained by manual building and AlphaFold3 combination. The atomic models of Hdf N-terminus (light green) and DNA (yellow) were directly generated by AlphaFold3. **(E)** Cut-open view of the intact structure of the contracted P1 with partial DNA. The inset shows the central cross-section of the head-connector in the contracted P1 with partial DNA. **(F)** Zoomed-in view of the inner surface around an icosahedral vertex to show the interactions among DarA, Hdf, DNA, and capsid in the contracted P1 with partial DNA. **(G)** Zoomed-in view of density maps (transparency) of Dar proteins and DNA in the contracted P1 with partial DNA superimposed on its atomic models. The color codes are identical to that in Fig 1D. **(H)** Zoomed-in view of the inner surface around an icosahedral vertex in the contracted P1 without DNA, DarA and Hdf extracted from the red box in panel A. **(I)** Organization of P1 genome segment. The color codes are applied to panels B-H.

Extended P1 was purified from *E. coli* strain K12 for cryo-EM data collection. The cryo-EM images revealed the presence of some tail-contracted particles among the extended P1 particles (Figs 1A and S1A). It was further observed that the DNA within partially contracted P1 particles was not completely released, thus classified as the contracted P1 with partial DNA. In contrast, a small number of particles had completely ejected their DNA accompanied by the capsid rupture or deformation, and was classified as the contracted P1 without DNA (Figs 1A and S1A). A total of 62,870 extended particles, 8,057 contracted particles with partial DNA, and 750 contracted particles without DNA were collected from 7,732 cryo-EM micrographs. Using icosahedral reconstruction method, we obtained an icosahedral structure of the extended P1 particle at a resolution of 4 Å [35]. By employing local reconstruction method [36,37], we improved the resolution of the 3- and 5-fold regions of the head to 3.6 Å (S1B Fig). Using the symmetry-mismatch reconstruction [38], the structure of the head-connector complex of P1 was resolved to a resolution of 8 Å (S2A Fig). We next performed the local refinement and reconstruction method [36,37] to further improve the resolution of the connector complex to 3.5 Å (S1B Fig). The baseplate was reconstructed by using the RELION [39] and cryoSPARC V4.6.0 [40] software, generating a resolution of 3.2 Å (S1B Fig). The density maps enabled us to build the atomic models for the major capsid protein (MCP) gp23, N-terminus of DarA, C-terminus of Hdf, portal protein Prt, adaptor protein PmgC, stopper protein gp7, tube initiator protein Tub, tube-linking protein PmgG, hub protein gp6, spike proteins (gp5 and UpfC), plug protein gp26, baseplate wedge protein 1 (BW1) PmgA, baseplate wedge protein 2 (BW2) gp16 and baseplate wedge protein 3 (BW3) Bp1A (S2B, S2C and S3 Figs). However, the C-terminus of DarA, the N-terminus of Hdf, tape measure protein (TMP) Sit, tripod UpfB, and fiber could not be resolved, possibly due to their flexibility. The structural resolution was determined based on the Fourier shell correlation criterion with a cut-off at 0.143 (S1 Table), according to the “gold standard” method.

The extended P1 is constituted by a DNA-containing head, a connector complex, a long non-contractiled tail, and a simple baseplate (Fig 1B). The capsid is composed of 775 copies of the MCP gp23, organized into 11 pentons, 60 center-hexons (C-hexons) and 60 edge-hexons (E-hexons) corresponding to a triangulation number of 13 (Fig 1B). Fifty-five pairs of Dar proteins, designated as DarA and Hdf, are attached at the junction of each penton and E-hexon on the capsid’s inner surface, where they interact with the DNA (Figs 1B–1D, and S4). The connector complex, connecting the tail to the head, consists of a dodecameric portal, a dodecameric adaptor, and a hexameric stopper (Fig 1B). Based on our previous work [34], the tail is composed of a hexameric tail terminator, 53 stacked hexameric rings of tail sheath gp22, and 52 stacked hexameric rings of tail tube BplB (Fig 1B). The baseplate constitutes the most complex module in P1, comprising 10 proteins: a hexameric tube initiator (Tub), a hexameric tube-linking protein (PmgG), a trimeric hub (gp6), a spike complex (trimeric gp5 and monomeric UpfC), three trimeric tripod (UpfB), TMP (Sit), six heterotetrameric wedges (each consisting of one BW1, one BW2 and two BW3), six-fold symmetric plug (gp26) and six fibers (Fig 1B).

Using the aforementioned methods, the structures of the 5-fold region and connector complex of the contracted P1 with partial DNA were reconstructed at resolutions of 4 Å and 4.5 Å, respectively (Figs 1E–1G and S1B). The density maps enabled us to build the atomic models for the N-terminus of DarA, the C-terminus of Hdf, portal Prt, adaptor PmgC, stopper gp7, and terminator gp24 (S5A and S5B Fig). Additionally, due to the capsid rupture or deformation, we were only able to box and reconstruct the structure of the 5-fold region of the contracted P1 without DNA at a resolution of 7.2 Å (Fig 1A and 1H). Local resolution maps for the P1 in all states are present in S6 Fig, generated with ResMap [41] and colored using ChimeraX [42]. In the contracted P1 with partial DNA, the external tail sheath underwent contraction towards the connector complex, thereby exposing the inner tail tube, and the baseplate was not observed. Although TMP and some DNA had been ejected, 55 DarA-Hdf pairs and residual DNA were still anchored within the head (Figs 1E and S5A). In the contracted P1 without DNA, all Dar proteins and DNA were ejected, accompanied by the capsid rupture or deformation (Figs 1A, 1H and S1A). Based on these observations, we speculate that the derivation of the contracted P1 maybe the interaction between the extended particles and the co-purified bacterial debris, thereby triggering tail contraction and DNA ejection in the purification process. Regarding the detachment of the baseplate in the contracted P1, we hypothesize that the mechanical stress from high-speed centrifugation could be a contributing factor. A similar phenomenon has been observed in phages Mu [43], XM1 [44] and T5 [45]. The phenomenon of the coexistence of the contracted P1 with DNA and without DNA has been reported in previous P1 studies [46], indicating that various contraction states naturally occur during the P1 life cycle.

### Structure of the icosahedral capsid

**Fig 2:**
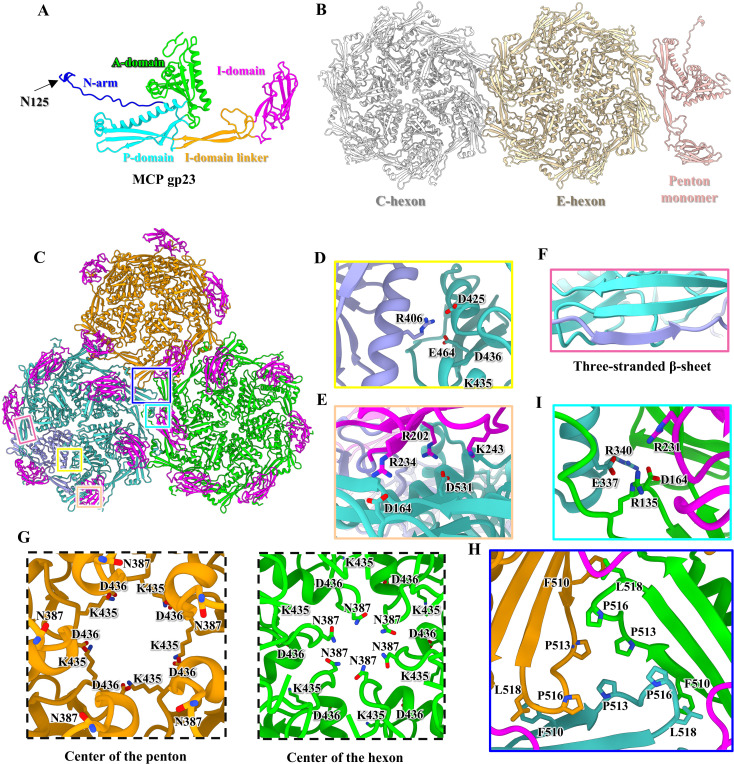
Structures of the major capsid protein gp23 of P1. **(A)** Ribbon model of the MCP gp23 shown in five domains. **(B)** Ribbon model of an asymmetric unit of the icosahedral capsid. Color codes are identical to that used in Fig 1B. **(C)** Top view of the capsomeric interactions along the three-fold and quasi-three-fold axis. One penton and two hexons are colored orange, green, and light sea green, respectively, except for an MCP colored in medium purple. The I-domains of all MCPs are colored magenta. **(D-F**, **H** and **I)** Zoomed-in views of the box regions in panel C to show the interactions in the intra- and inter-capsomeres, including hydrogen bonds and salt bridges **(D**, **E** and **I)**, β-sheet augmentation **(F)**, and hydrophobic pocket **(H)**. **(G)** Structural comparison of the centers of penton (left) and hexon (right).

The MCP gp23 in P1 adopts a canonical HK97-like fold [47], and it also contains an additional insertion domain that exhibits structural similarity to domains in T4 [48], N4 [49] and phiKZ [50] (S7A Fig). According to the nomenclature of the MCP gp24 in T4, gp23 can be divided into five domains (Fig 2A): an N-arm, P-domain, I-domain linker, I-domain, and A-domain. The N-terminus 124 residues of gp23 were not resolved in our density map (Fig 2A), which is consistent with a biochemical analysis indicating that the N-terminus of gp23 undergoes proteolytic processing by the predicted prohead protease Pro during capsid morphogenesis [25]. In phages HK97 [51] and T5 [52], their N-termini are hydrolyzed and are proposed to function as the scaffold protein. Using AlphaFold3 to predict the full-length structures of MCPs among P1, HK97 and T5, we find that their N-termini show a coiled-coil, heptad-repeat pattern, exhibiting a structural similarity to the scaffold protein of P22 [53] (S7B Fig). Therefore, the N-terminus of gp23 is hypothesized to function as the scaffold protein of P1, as predicted in the previous reports [24]. Notably, the icosahedral capsid of the majority of T = 13 phages, including T5 [54], Mic1 [55] and HVTV-1 [56], with diameters of approximately 910, 920 and 880 Å, respectively, are smaller in size than the capsid of P1 (~980 Å diameter) (S7C Fig). The MCPs of T5, Mic1 and HVTV-1 lack an I-domain, while the β-sheet-rich I-domain of P1 gp23 protrudes outward from the capsid surface, which is likely to contribute to the larger head size (S7C Fig). It is therefore postulated that the larger P1 head may accommodate the substantial mass of Dar proteins, such as the ~ 2,000 amino acids DarB protein, which is predicted to be present in ~45 copies [22].

Superimposition of the 13 gp23 monomers from an asymmetric unit reveals significant conformational changes in the N-arms, I-domain linkers, and I-domains (Figs 2B, S8A and S8B), which result in the formation of three distinct capsomeres: P-hexon, C-hexon, and penton (S8C Fig). The MCP monomers in both penton and hexon exhibit identical structural arrangements and interaction patterns (Fig 2C), where the central interactions are primarily formed by two adjacent A-domains (Fig 2D), the peripheral interactions are mediated by the P-domains, and adjacent I-domains and N-terminuses (Fig 2E and 2F). However, the interactions at the centers between the penton and hexon differ significantly (Figs 2G and S8B). Despite the absence of cement proteins at the capsomere interfaces [57], extensive non-covalent interactions occur around the three-fold and quasi-three-fold axis. Hydrophobic residues on the P-domain of each capsomere cluster together, forming a large hydrophobic pocket to stabilize the capsid (Fig 2H). Furthermore, the I-domain of a MCP monomer and the N-terminus of an adjacent MCP monomer from a capsomere interact with the P-domain from an adjacent capsomer, providing additional stability (Fig 2I). Similar inter-capsomere stabilization mediated by an insertion domain occurs in A-1(L) [58] and R4C [59].

### Dar proteins DarA and Hdf

**Fig 3:**
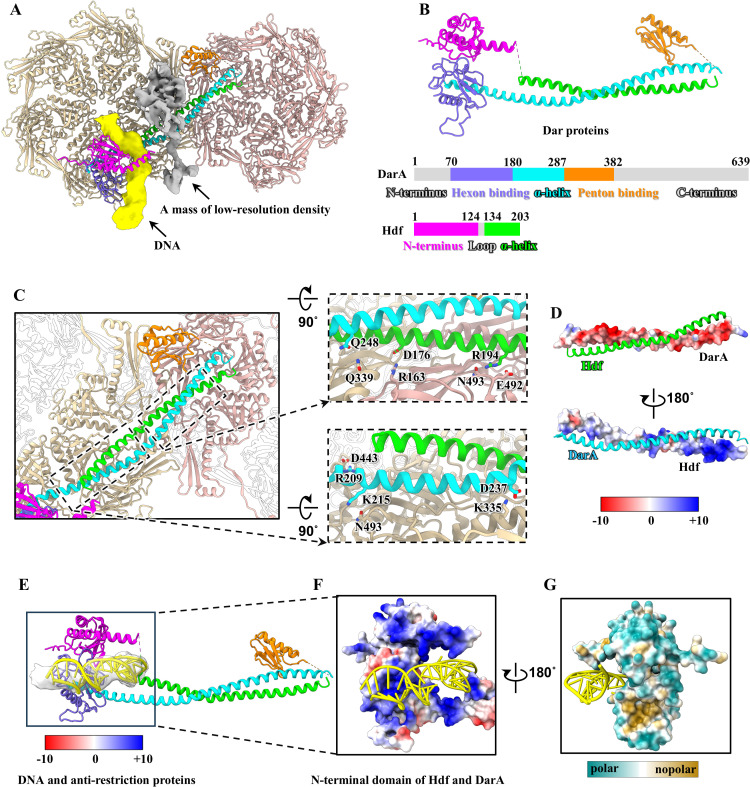
Structures of the Hdf and DarA in P1. **(A)** Interactions between Dar proteins, DNA, and capsid. Color codes are identical to that used in Fig 1B, except for Hdf and DarA colored according to their domains. The color code is applied to panels A-E. **(B)** Ribbon model of the Dar proteins, colored according to their domains. The N-terminus of Hdf is predicted by AlphaFold3. **(C)** Zoomed-in views of the interactions among the capsid and the α-helical domains of DarA and Hdf. **(D)** Electrostatic potential surfaces of α-helical domains between Hdf and DarA. **(E)** Interactions among DNA and N-terminal domain of Hdf and DarA. **(F, G)** Positively charged inner surface **(F)** and hydrophilic outer surface **(G)** of the N-terminal domain of Hdf and DarA.

We built the atomic model for the Hdf C-terminus (residues 135–203 out of the 203 residues) (S3 Fig), designated the α-helix domain (Fig 3A and 3B). This domain interacts with the capsid inner surface via salt bridges and hydrogen bonds (Fig 3C). The N-terminus (residues 1–124) of Hdf could not be modelled due to its flexibility. AlphaFold3 prediction [60] suggests that the Hdf N-terminus comprises two α-helices and a four-stranded β-sheet, connected to its C-terminus by a short loop (residues 125–134) (S9A Fig). A low-resolution density near the Hdf C-terminus, adjacent to DNA (Fig 3A), fits well with the predicted atomic model of the Hdf N-terminal domain (S9B Fig). Furthermore, the biochemical experiment had indicated that, in contrast to the DarA, Hdf does not undergo proteolytic processing [24]. Consequently, we assign this density may belong to the Hdf N-terminus. Furthermore, around the low-resolution density, we observed a density fragment that exhibits a characteristic double-helix structural feature, analogous to previously published low-resolution maps of phage DNA, such as T5 [45] and A4 [61]. Additionally, this density fragment also fits well with established DNA atomic model (S3 Fig). Therefore, we hypothesize that the density fragment may belong to the DNA.

We modelled DarA from residues 70–382 (out of 639 residues) (S3 Fig). The C-terminal domain (residues 383–639) could not be resolved possibly due to its flexibility. Considering the interaction environment with Hdf, capsid, and DNA, each DarA monomer can be divided into three domains (Fig 3A and 3B): an N-terminal hexon binding domain, an α-helix domain, and a penton binding domain. The hexon binding domain closely interacts with the hexon inner surface via a long loop (residues 102–116) (S9C Fig). Density for the distal N-terminus (residues 1–69) was not visible (S9C Fig), consistent with biochemical experiments that a ~ 9 kDa fragment (~75–90 amino acids) of DarA is cleaved by P1 prohead protease Pro during capsid morphogenesis [62,63]. We speculate that residues 1–69 undergo proteolytic cleavage during capsid formation. The α-helix domain is anchored to the capsid inner surface via salt bridges and hydrogen bonds (Fig 3C). The penton binding domain is connected to the penton inner surface via two loops (residues 321–326, 342–346) (S9C Fig). Comparing our DarA structure with the full-length AlphaFold3 prediction, the hexon binding domain and the penton binding domain are quite similar, but the predicted C-terminus shows a comparatively low Predicted Local Distance Difference Test (pLDDT), presumably due to its flexibility (S9A and S9D Fig). We also observed a mass of low-resolution density surrounding a penton binding domain and α-helix domain (Fig 3A). However, its atomic model could not be built due to the low resolution, and thus the identity of this protein could not be determined.

Genome sequencing reveals the presence of Dar proteins, including DarA and Hdf homologs, in the head of P1-like phages such as *Salmonella* phage SJ46 [31], *E. coli* phages RCS47 [32] and JL22 [33]. AlphaFold3 structural predictions and sequence analysis of their DarA and Hdf demonstrate a high degree of structural similarity and sequence identity between P1 and P1-like phages (S10 Fig), suggesting common evolutionary origins. This finding implies that the Dar proteins, which is located inside the head, are likely a conserved feature among P1-like phages. These proteins are hypothesized to function in defense against R-M systems in hosts such as *E. coli* and *Salmonella.* To date, the in situ structure of Dar proteins has only been resolved for P1, thereby emphasising their significance for future structural and functional studies.

### Interactions among DarA, Hdf, DNA, and capsid

On the capsid’s inner surface, 55 DarA-Hdf pairs are anchored to the junction of each penton and E-hexon and simultaneously interact with the DNA, demonstrating a unique interactional feature among DarA, Hdf, DNA, and capsid (Fig 3A). DarA and Hdf primarily interact through their α-helical domains, adopting a parallel α-helix bundle conformation stabilized by complementary electrostatic potential (Fig 3D). Each α-helix bundle forms extensive contacts with the capsid inner surface via hydrogen bonds and salt bridges (Fig 3C), thereby reinforcing the stability of the junction of penton and E-hexon. It was consistent with our experiments that in the contracted P1 without DNA, the absence of Dar proteins leads to the capsid rupture or deformation (Fig 1A and 1H) and the probability of the rupture or deformation in this state is higher than that of other DNA-ejected phages [37,45]. The N-terminal domains and α-helical domains of Hdf and DarA together form a complex (Fig 3E). The DNA-facing side of the complex is rich with positively charged residues and interacts with the DNA (Fig 3F). As with the reported phages [64,65], it is possible that positively charged small molecules such as spermine in DarA-Hdf pairs may assist in neutralizing the negative charge of dsDNA packaged within P1, thereby reducing the internal pressure and maintaining phage stability. Conversely, the outer surface of the complex is enriched with hydrophilic residues (Fig 3G), which may be necessary to maintain the solubility of the DNA-protein complex and prevents aggregation in the hydrophilic host cytoplasm.

Biochemical analysis indicated that each P1 particle contains approximately 100 copies of DarA, and 400 copies of Hdf [22]. However, we observed only 55 DarA-Hdf pairs bound at the junction of each penton and E-Hexon. This finding indicates that only a proportion of DarA and Hdf are anchored to the capsid and involved in interacting with DNA located near the capsid surface. The remaining components may be associated with located toward the interior center of the capsid or may exist in different conformations. In the contracted P1 with partial DNA, a portion of the density of 55 DarA-Hdf pairs was still observed on the capsid’s inner surface and interacted with the DNA (S5A Fig). Detailed comparisons of the extended P1 and the contracted P1 with partial DNA reveal density reductions in several head structures, including the low-resolution densities and the penton binding domain of DarA (Figs 1C, 1D, 1F, 1G and 3A). The exact causes of these density reductions require further investigation. In the contracted P1 without DNA, all Dar proteins and DNA were ejected (Fig 1H). We propose that, during the process of tail contraction, the DNA and Dar proteins, which are located toward the interior center of the capsid, first undergo release, followed by the release of 55 DarA-Hdf pairs and DNA targeted on the capsid.

### Structure of the portal-adaptor-stopper complex

**Fig 4:**
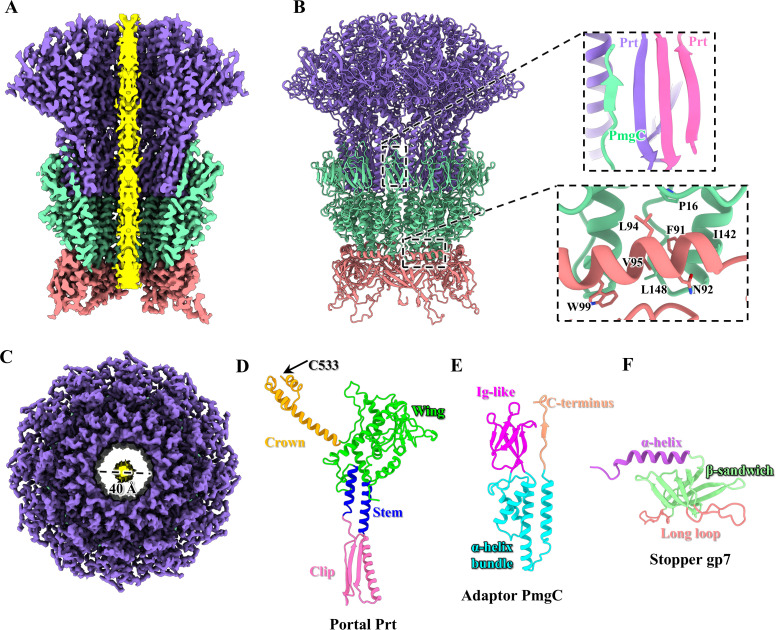
Structures of the connector complex of the extended P1. **(A**, **B** and **C)** Cut-open **(A)**, side **(B)** and top **(C)** views of the connector complex of the P1. Color codes are identical to that used in Fig 1B. The top inset shows a zoomed-in view of the interactions between an adaptor monomer and two adjacent portal monomers, forming a four-stranded β-sheet. The bottom inset shows a zoomed-in view of the α-helix domain of the stopper capping the α-helix bundle domain of the adaptor by forming a huge hydrophobic pocket. **(D**, **E** and **F)** Ribbon models of the portal protein Prt **(D)**, the adaptor protein PmgC **(E)** and the stopper protein gp7 **(F)**, colored according to their domains.

As with the majority of myophages [66,67] and siphophages [59,68], the head-to-tail connector of P1 consists of a dodecameric portal, a dodecameric adaptor, and a hexameric stopper, providing the interaction interface for tail attachment (Fig 4A–4C). The portal contains 12 copies of protein Prt, which exhibits significant structural similarity to portals in other tailed phages [69]. Following the protein nomenclature, each Prt monomer is comprised of four domains (Fig 4D): a C-terminal crown domain, a wing domain, a stem domain, and a clip domain. Notably, the density for the distal C-terminus (residues 534–569) was absent, probably due to flexibility (Fig 4D). The P1 adaptor comprises 12 copies of protein PmgC, which exhibits a conserved structure among tailed phages [68,70]. Each PmgC monomer consists of three domains (Fig 4E): a C-terminal domain, an α-helix bundle domain, and an Ig-like domain. The C-terminal domain of each adaptor monomer is anchored to the clip domains of two adjacent portal monomers, forming a four-stranded β-sheet (Fig 4B). It is possible that after the binding of the adaptor on the portal, the density of the clip domain is more visible, suggesting that the interface adaptor/portal adopted a unique conformation that might contribute to the stability of the complex. Six copies of the stopper protein gp7 form a hexameric ring which connects to the dodecameric adaptor. Structurally analogous to stoppers in siphophages [59,68] and myophages [66,67], gp7 comprises three domains (Fig 4F): an N-terminal α-helix domain, a β-sandwich domain, and a long loop domain. The six α-helix domains form a circular ring that caps the adaptor’s α-helix bundle domain via a large hydrophobic pocket (Fig 4B), as also observed in other siphophages [71,72], and myophages [67,73]. The long loop domain of gp7 docks onto the hexameric tail terminator protein gp24 (S11A Fig). Structural analysis reveals that the interfaces between the portal-adaptor-stopper connector and the tail terminator are complementary in shape and charge, which enables sequential joining and efficient assembly of the P1 (S11B Fig). The structural comparison between the extended and contracted states of P1 indicates that the connector complex and tail terminator remain unchanged, with the exception of the C- and N-terminus of the tail terminator (S12 Fig). The C- and N-terminus of the tail terminator became invisible due to the release of the TMP, which allows more flexibility in that area, while the structure of the protein itself remains unchanged. Notably, the connector complex and tail terminator form a channel with a minimum inner diameter of approximately 40 Å (Fig 4C). Structural comparisons of the minimum diameters of the connector complex among moyphages P1, Mu [43], E217 [66], Pam3 [67] and phiTE [74] that reveals that the minimum diameters of the connector complex of these myophages are smaller in size than that of P1 (S13 Fig). This may provide an indirect evidence that the Dar proteins and DNA form a huge complex and are released together, thus the connector complex in P1 also need to evolve wider channels than those of other myophages, thereby providing sufficient space for both DNA and Dar proteins to pass during infection.

### Structure of the simple baseplate

**Fig 5:**
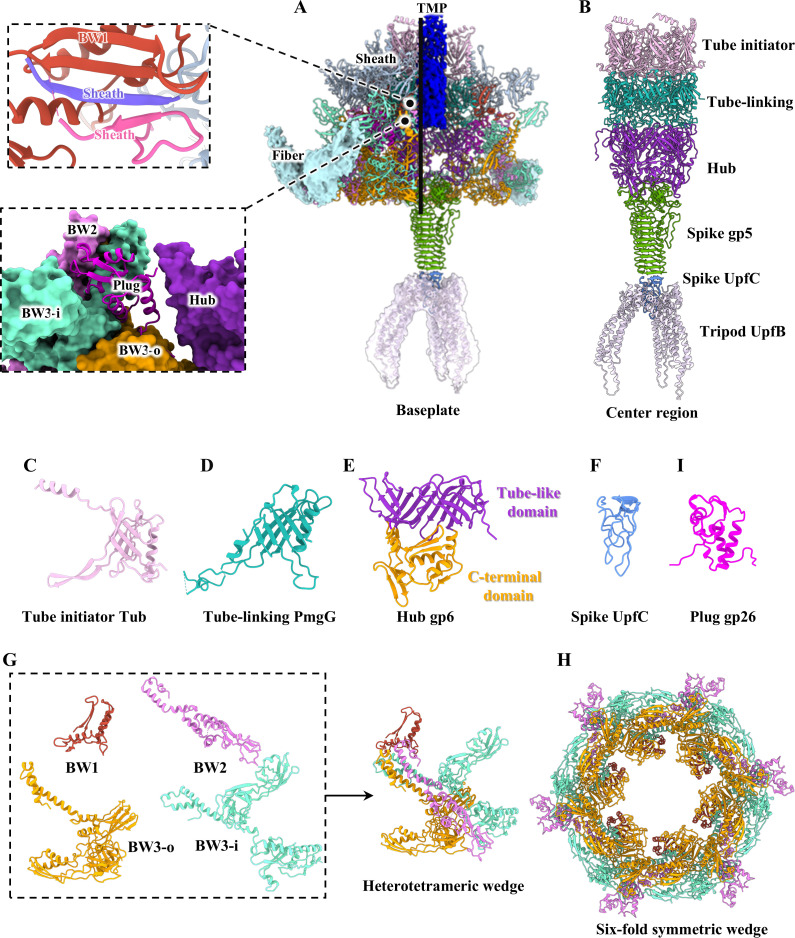
Structures of the simple baseplate in extended P1. **(A)** Side (left) and cut-open (right) views of the ribbon models of the simple baseplate. The top inset shows a zoomed-in view of the interaction between a BW1 monomer and two adjacent sheath monomers, forming a four-stranded β-sheet. The bottom inset shows a zoomed-in view of the interactions among plug, hub and wedge. The color coding is identical to that used in Fig 1B. The tripod and fibers are shown in density maps. **(B)** Ribbon model of the center region of the baseplate. **(C-F** and **I)** Ribbon models of the tube initiator Tub **(C)**, tube-linking PmgG **(D)**, hub gp6 **(E)**, spike UpfC **(F)** and plug gp26 **(I)**. The hub protein gp6 is colored according to its domains. **(G)** Ribbon models of BW1, BW2, BW3-i and BW3-o in the heterotetrameric wedge. **(H)** The bottom view of the ribbon model of the six-fold symmetric wedge.

The P1 simple baseplate exhibits a structurally conserved architecture across myophages [43,66,67] and contractile injection systems (CISs) [75,76]. According to the protein nomenclature for the Mu baseplate [43], the P1 baseplate is divided into two major regions (Fig 5A and 5B): the central and peripheral regions. The central region is constituted by the tube initiator (Tub), tube-linking protein (PmgG), hub (gp6), spike (gp5 and UpfC), tripod UpfB and TMP (Sit). The peripheral region is comprised of the wedge (PmgA, gp16 and Bp1A), plug gp26 and fiber. Previous studies have demonstrated that contraction of the tail sheath results in physical separation of the peripheral region from the central region [66,76].

In the central region of the P1 baseplate, both the tube initiator Tub and the tube-linking protein PmgG exhibit similar tube-like folds with the tube BplB, forming a six-fold symmetric tube-ring (Figs 5C, 5D and S14A). The assembly of the tail tube BplB in P1 is initiated by the tube initiator, while tube-linking protein PmgG primarily mediates the symmetry mismatch between the six-fold symmetric tube ring and the three-fold symmetric hub. The P1 hub gp6 is composed of a tube-like domain and a C-terminal domain (Fig 5E), which displays a structural similarity to those found in the reported myophages [43,67] and CISs [75,76]. The tube-like domains of three gp6 monomers are organized into a pseudo-six-fold symmetric tube-ring, which is topologically identical to that observed in the tube BplB (S14A Fig). The trimeric spike gp5 and monomeric UpfC, assembled into a spike complex, exhibit a striking structural similarity to the trimeric gp5 and monomeric gp5.4 of the spike complex in T4 [77] (Figs 5B, 5F and S14B). Gp5 in T4 contains a lysozyme domain [78], but gp5 in P1 does not, suggesting that the transmembrane mechanism of P1 is different from that of T4 (S14B Fig). Notably, P1 encodes a novel three-trimeric tripod UpfB, which is anchored to gp5.4 of the distal tail (Fig 5B). Despite only a medium-resolution map of the UpfB was obtained, the AlphaFold3-predicted trimeric structure can fit well into the density (Fig 5A). It is speculated that the spike gp5, UpfC and UpfB together form a membrane-piercing apparatus located at the tail end, which may play a significant role in transmembrane function. While the TMP Sit could not be resolved due to its asymmetric assembly and inherent flexibility within the tail cavity, a low-resolution density was observed inside the central region of the baseplate (Fig 5A). Sequence analysis revealed that Sit contains a soluble lytic transglycosylase domain [24], that specifically degrades peptidoglycan in the bacterial cell wall. This function may disrupt cell-wall integrity locally to create physical channels that facilitate subsequent transmembrane processes, such as macromolecular transport or transmembrane-protein assembly.

In the peripheral region of the P1 baseplate, the wedge comprises six heterotetramers, each composed of one BW1 PmgA monomer, one BW2 gp16 monomer, and two conformers of BW3 Bp1A monomers (referred to as BW3-i and BW3-o) (Fig 5G and 5H). As has been previously reported for other myophages [43,66] and CISs [75,76], the N-terminal regions of BW2, BW3-i and BW3-o in a heterotrimer are arranged in an approximately parallel architecture forming a core helical bundle, while the remaining heterotrimer folds into a canonical trifurcation unit (Fig 5G). Each BW1, as the sheath initiator, is located at the top of the core helical bundle within the wedge and is docked at the tube-linking protein PmgG (Fig 5A and 5G). The N-terminus of one monomer and the C-terminus of the adjacent monomer from the first layer of the sheath gp22 interact with the BW1, forming a conserved four-stranded β-sheet to initiate sheath assembly, as seen in Mu [43], phiTE [74] and Pam3 [67] (Fig 5A). The BW2 and BW3-i are located at the periphery of the baseplate, while the BW3-o oligomerizes into the inner ring of the peripheral region in the baseplate and is in close proximity to the C-terminus of the hub (Fig 5A and 5H). The six-fold symmetric plug gp26 acts as a binding agent, facilitating the attachment of the wedge to the outer surface of the central region (Fig 5I). A comparison of myophages P1 and E217 [66], Pam3 [67] and XM1 [44] reveals both structural differences and conserved functional characteristics (S14C Fig). Six fibers with weakly densities are anchored to the wedge protein gp16; however, due to their flexibility, they could not be resolved at near-atomic resolution (Fig 5A).

## Discussion

In this study, we determined the structures of P1 in three states at near-atomic resolutions, and identified structural proteins for the P1, including the head, connector complex, and baseplate. The structural comparison of P1 with other myophages [43,66,67], as well as CISs [75,76], reveals that the majority of proteins of the head, connector complex, and baseplate are conserved, while Dar proteins inside the head of P1 and P1-like myophages are the notable exceptions.

During assembly of numerous tailed phages, the portal protein initiates assembly. MCP monomers first form a spherical procapsid shell, accompanied by the co-packaging of scaffold proteins [79,80]. In some phages, like HK97 [47] and T5 [81], their MCP N-termini function as an internal scaffolding domain. Whether as individual proteins or N-terminal domains, these scaffolding components establish specific interaction networks within the head, guiding MCPs to assemble at defined spatial angles, thereby precisely modulating capsid curvature and size. Subsequently, the exit of scaffolding proteins or the hydrolysis of the MCP N-terminus provide the conditions for the expansion of the procapsid shell. DNA translocation is initiated through the portal complex into the interior of the capsid [54,82]. DNA packaging occurs concurrently with capsid expansion [47,54,82], ultimately driving the procapsid expanding into the mature icosahedral capsid [53,83]. Although the vast majority of phages naturally produce a single head size [53,84], some can alter head sizes under specific circumstances. For instance, T4 requires specific mutations for head size variation [85,86]. When 80α serves as a helper phage for *Staphylococcus aureus* pathogenicity islands (SaPIs), such as SaPI1, its capsid assembly pathway is redirected, resulting in a transition from T = 7 to T = 4 icosahedral heads [87]. Notably, P1 phage possesses the unique ability to spontaneously form three distinct head sizes: “normal” (~980 Å diameter), “small” (~650 Å diameter), and “minute” (~470 Å diameter) [88]. Despite belonging to different systems, both P1 and 80α can produce smaller heads without artificial intervention. Based on their biochemical and structural studies, we propose P1’s small head mechanism, and systematically compare these two systems.

In the 80α system, capsid size modulation depends on proteins CpmA (gp7) and CpmB (gp6) encoded by SaPI1 [87,89]. CpmB functions as a scaffold protein, competing with 80α’s native scaffold proteins for the binding sites on MCPs, while CpmA maybe serve as an accessory factor that facilitates CpmB function [90]. Together, they modulate inter-MCP spatial angles to control capsid curvature and size, ultimately producing T = 4 small capsid. In P1, MCPs oligomerize into the procapsid, perhaps accompanied by internal packaging of DarA-Hdf pairs [22]. Although P1 lacks an individual scaffold protein, MCP N-terminus assumes the primary role of the scaffold protein [24]. Biochemical evidence confirms that both the MCP N-terminus [25] and DarA N-terminus [62,63] are proteolytically cleaved by the protease Pro. Notably, the cleavage sites of MCP and DarA N-termini are spatially adjacent (S9C Fig). Based on the model of exogenous proteins involvement in 80α assembly, we hypothesize that the N-termini of the MCP and DarA cooperatively provide scaffolding function, while Hdf may serve a role analogous to CpmA in 80α, assisting DarA function during this process.

However, unlike the 80α system where CpmB induces small head formation through competitive MCP binding sites [90], the P1 system may exhibit variable binding probabilities between the DarA N-terminus/MCP N-terminus and MCP binding sites, ultimately resulting in the formation of heads with varying sizes. Experimentally, parental P1 virions produce ~ 82% normal heads and ~18% small heads [22]. Deletion of either DarA or Hdf results in a significant reduction in the proportion of normal heads, from ~80% to ~15% [22]. We therefore hypothesize that the absence of the DarA-Hdf pair has a higher probability of disrupting the scaffolding network, and altering the internal interaction patterns within the procapsid, therefore favoring the production of small heads. Notably, in contrast to 80α where CpmA/CpmB are proteolytically cleaved or excluded following maturation [90], the DarA-Hdf pair is retained in mature P1, with their α-helix domains forming tight interactions at the junction of the penton and E-hexon on the capsid’s inner surface. Comparative analysis of 80α and P1 reveals that both systems modulate capsid size by introducing proteins with additional scaffolding functions that alter the scaffolding network environment. Conversely, the procapsids of most phages lack such additional scaffold components [47,53,84], relying solely on the scaffold proteins or MCP N-termini for assembly, thus typically producing heads of a single size. These additional scaffold components capable of size regulation may be universally present in phage systems that can form multiple head sizes. The comparative study of P1 and 80α provides critical insights into the molecular mechanisms underlying head size diversification in phages.

As demonstrated in previous biochemical studies, all Dar proteins are known to incorporate sequentially, with Hdf and DarA being packaged in an early manner, followed by the ordered incorporation of DdrA, DdrB, DarB and Ulx [22]. Deletion of Hdf and DarA results in the exclusion of other Dar proteins (DarB, DdrB, Ulx and DdrA) from packaging [22]. Although the assembly timing for other Dar proteins is unclear, our structure suggests that they may adsorb around Dar-Hdf pairs during the subsequent packaging stage, thereby potentially forming a large DNA-interacting complex near the Hdf and DarA. Consequently, although we observed a mass of low-resolution density surrounding a penton binding domain and α-helix domain of DarA, it could not be confidently attributed to any particular component. During the infection process, as has been previously reported for other myophages [44,66,91], P1 releases the energy through sheath contraction, providing sufficient force for the tail to penetrate the host cell membrane. The ~ 40 Å channel formed by the connector complex and tail terminator allows for the passage of both DNA and Dar proteins. DNA and Dar proteins located toward the interior center of the capsid may be released first, followed by 55 DarA-Hdf pairs and DNA complex attached to the inner surface of the capsid. Upon successful ejection, Hdf and DarA protect DNA against EcoA and StySA type I R-M systems [22,24], while their hydrophilic molecules on outer surface maybe necessary to maintain the solubility of the DNA-protein complex and prevent aggregation in the cytoplasm. In conclusion, the close interactions among Hdf, DarA, DNA, and capsid revealed by our structures of three states of P1, allow us to propose a molecular mechanism for the function of these Dar proteins during phage assembly, infection and DNA ejection. It is hypothesized that this mechanism is conserved among P1-like phages.

## Materials and methods

### Production and purification of phage P1

The extended P1 was prepared using an established method [34]. *E. coli* strain K12 (ATCC 25404) was cultivated in LB liquid medium (5 g Yest extract, 10 g tryptone, and 10 g/L NaCl) at 37 °C for 4 h. The P1 phage (ATCC 25404-B1) was then inoculated into the bacterial culture at 37 °C for 4 h. After the bacterial lysis, the P1 phage in the supernatant was separated and collected by using low-speed centrifugation at 4000 × g for 30 min at 4 °C. The supernatant was precipitated with 1 M NaCl and 10% PEG8000, and stored at 4 °C overnight. The precipitated particles were resuspended in phage buffer (50 mM NaCl, 10 mM Tris-HCl, 5 mM MgCl2, and pH 7.4) and purified further by CsCl gradient centrifugation using densities of 1.5 g/mL and 1.4 g/mL at 135,000 × g at 8 °C for 2 h. The P1 phage band was then extracted and dialyzed against phage buffer at 4 °C overnight. Finally, purified P1 phages were stored on ice-water for cryo-sample preparation.

### Data acquisition and image processing

The purified P1 phage aliquot (3 µL) was pipetted onto amorphous nickel-titanium alloy grids, which were glow-discharged for 30 s. The grids were loaded into an FEI Vitrobot with humidity of 100%, temperature of 8 °C, and blotting time of 4.0 s. The grids were then plunged into a solid-liquid ethane and transferred to liquid nitrogen. The cryo-EM data was collected using a Titan Krios G3i microscope with 300 keV, equipped with a K3 summit direct electron detector. The FEI EPU software automatically collected images of the phage particles at 53,000 × magnification, resulting in a pixel size of 1.36 Å. The full dose of each movie was approximately 30 e^-^/Å^2^. A total of 7,732 movies were collected with each movie stack consisting of 32 image frames. The defocus values and astigmatism of each image were calculated using GCTF [92] packaged in RELION [39]. Using the ETHAN software [93], a total of 62,870 extended particles, 8,057 contracted particles with partial DNA, and 750 contracted particles without DNA were selected. The icosahedral head of P1 was reconstructed to a resolution of 4 Å using our programs [35,94] based on the common-line algorithm [95,96]. Using the local reconstruction method [36,37], the 3- and 5-fold regions of the head in the extended P1 were next improved to resolutions of 3.6 and 3.6 Å, respectively.

### Symmetry-mismatch and local reconstruction

The asymmetric structure of myophage Mu [43], filtered to 60 Å resolution, was used as the initial model. Using the symmetry-mismatch reconstruction method [36,38], we reconstructed the 3D asymmetric structure of the head-connector complex in the extended P1 at a medium resolution of 8 Å. The reconstruction step is described below: (1) For each two-dimensional (2D) particle image, we searched for the asymmetric orientation from 60 equivalent orientations of the icosahedral orientation based on an initial model. (2) Using the latest orientation, a new 3D asymmetric structure was reconstructed without imposing any symmetry. (3) The above steps (1) and (2) were iterated in approximately 70 rounds until the orientations of all particle images remained unchanged. Using the local refinement and reconstruction method [36,37], the structure of the connector complex of the extended P1 was improved to a resolution of 3.5 Å by imposing 12-fold symmetry. By using the same method, the structures of the 5-fold region and the connector complex of the contracted P1 with partial DNA were reconstructed at resolutions of 4 Å and 4.5 Å, respectively; we only boxed each vertex of the icosahedral head in the contracted P1 without DNA and reconstructed the structure of its 5-fold region at a resolution of 7.2 Å.

The local reconstruction of the baseplate in the extended P1 was performed using the RELION 3.1.1 [39] and cryoSPARC V4.6.0 [40] software. A total of 37,799 baseplate particles were manually selected with a box size of 300 × 300 pixels. 2D and 3D classification were next performed to the removal of irrelevant particles. After selection, 30,339 particles were used for 3D refinement to determine the structure of the baseplate at a resolution of 3.7 Å, by imposing six-fold symmetry. Subsequently, the baseplate dataset was converted into the cryoSPARC for further processing. All particle images perform 3D classification using the WALC (Walking ALignment and Classification) procedure to separate different classes, and then to align selected classes. The resulting particles were performed a 3D refinement to obtain the structure of the baseplate at a resolution of 3.2 and 3.7 Å, by imposing three-fold symmetry and without imposing the symmetry, respectively.

### Atomic modelling building and refinement

Based on our cryo-EM density maps of the head-tail in the extended P1, the atomic models of MCP gp23, N-terminus of DarA, C-terminus of Hdf, portal Prt, adaptor PmgC, stopper gp7, tube initiator protein Tub, tube-linking protein PmgG, hub protein gp6, spike proteins (gp5 and UpfC), plug gp26, BW1 PmgA, BW2 gp16 and BW3 Bp1A were manually built using the COOT software [97]. Additionally, the models of tripod UpfB and the N-terminus of Hdf were obtained using AlphaFold3. Based on density maps of the contracted P1, we built models for N-terminus of DarA, C-terminus of Hdf, portal Prt, adaptor PmgC, stopper gp7, tail terminator gp24. All atomic models were further refined using the real-space refinement method, implemented in Phenix [98]. The refinement and validation statistics are shown in S1 Table.

## Supporting information

S1 Fig Cryo-EM images and Fourier shell correlation curves of three states of P1. (A) Representative cryo-EM images of the extended and contracted P1. The contracted P1 with and without DNA was boxed black and red, respectively. (B) Estimated structural resolutions of the 5-fold region (3.6 Å), the 3-fold region (3.6 Å), the connector complex (3.5 Å), the baseplate (3.2 Å) imposing C3 symmetry and the baseplate (3.7 Å) imposing C1 symmetry in the extended P1, and the 5-fold region (4 Å), and the connector complex (4.5 Å) in the contracted P1 with partial DNA, as well as the 5-fold region (7.2 Å) in the contracted P1 without DNA.
(TIFF)

S2 Fig Quality of the cryo-EM density maps and atomic models from the head-tail in the extended P1. (A) Side views of the symmetry-mismatched structure of the head-connector complex in the extended P1. (B) Cut-open view of the intact structure of extended P1. DNA is manually removed to show the inner surface of head. Color codes are identical to that used in Fig 1B. (C) Ribbon models of almost all protein components from the head-tail and density maps (transparency) of partial protein components superimposed on their atomic models. All the atomic models were manually built, except for UpfB modelled by AlphaFold3.
(TIFF)

S3 Fig Structures of the Dar proteins and DNA. Density maps (transparency) of Dar proteins and DNA are superimposed on their atomic models. The color codes are identical to that in Fig 1D.
(TIFF)

S4 Fig Cross-sections of P1 extended head (top) and contracted head without DNA (bottom). Left panel shows the bottom view of 5-fold vertex structure of the head. Right panel shows cross-sections at various thicknesses of the 5-fold vertex of the head.
(TIFF)

S5 Fig Quality of the cryo-EM density maps and atomic models from the head-tail in the contracted P1. (A) Cut-open views of the head-tail in the contracted P1 with partial DNA. DNA is removed manually to show the inner surface of head. Color codes are identical to that used in Fig 1B. (B) Ribbon models of all protein components from the head-tube complex, and density maps (transparency) of partial protein components superimposed on its atomic models.
(TIFF)

S6 Fig Local resolution maps of the P1 from the head to the baseplate in all three states. (TIF)

S7 Fig Structural comparison of the P1 MCP gp23 with other phage MCPs. (A) Structural comparison between the P1 MCP and the HK97/T4 MCP (PDB ID: 1OHG/ 5VF3). (B) Structural comparisons of the scaffold domain or scaffold protein predicted by AlphaFold3 (copper colors) among P1, T5, HK97 and P22. (C) Top: Cut-open views of the density map of the icosahedral capsids, including P1, T5 (EMD-20125), Mic1 (EMD-9774) and HVTV-1 (EMD-2234). Bottom: Ribbon models of the MCP among P1, T5 (PDB ID: 6omc) and Mic1 (PDB ID: 6j3q).
(TIFF)

S8 Fig Structure of the MCP gp23 in P1. (A) Superimposition of the 13 MCPs, displayed in different colors. (B) Zoomed-in views of density maps (transparency) superimposed on atomic models of the hexameric and pentameric monomer (sticks). (C) Top and side views of ribbon models of the penton, E-hexon, and the C-hexon. Color codes are identical to that used in Fig 1B, except for a monomer of each capsomer colored in magenta.
(TIFF)

S9 Fig Structures of the Hdf and DarA in P1. (A) Ribbon models of the DarA and Hdf predicted by AlphaFold3. The Predicted Local Distance Difference Test (pLDDT) is shown in the color bar. (B) Density map (transparency) of Hdf superimposed on its atomic model modelled by AlphaFold3. (C) Zoom-in views of the interactions between DarA and capsid. Color codes are identical to that used in Fig 3A. (D) Comparison of the N-terminus of DarA from the hexon binding domain to the penton binding domain between our structure and the predicted structure.
(TIFF)

S10 Fig Comparison of structural similarity and sequence identity of Hdf and DarA among P1 and P1-like phages. All structures of Hdf and DarA in P1-like phages are predicted by AlphaFold3.
(TIFF)

S11 Fig Complementary electrostatic potential of the extended P1. (A) Cut-open view of ribbon models of the connector-tube complex (tube PDB ID: 8jan). Color codes are identical to that used in Fig 1B. (B) Electrostatic potential surfaces between two adjacent protein components among the connector complex and tail terminator. The top columns are oriented toward the tail, whereas the bottom columns are oriented toward the head. The electrostatic potential scale is shown in the color bar.
(TIFF)

S12 Fig Structural comparisons of the connector complex and the tail terminator in the extended and contracted P1. (A, D) Cut-open views of the density map from the connector complex to the tail in the extended (A) and contracted (D) P1. Color codes are identical to that used in Fig 1B. (B, C) Zoomed-in views of the box regions in panel A to show the interactions among the tail terminator-sheath (B) and tail tube-sheath (C) of the extended P1. (E) Zoomed-in view of the interactions between the tail terminator, tail tube and tail sheath of the contracted P1. (F) Structural comparisons of the portal, adaptor, stopper and terminator in the extended and contracted P1.
(TIFF)

S13 Fig Structural comparisons of the connector among Mu (EMDB-62359), Pam3 (EMDB-34678 and EMDB-34679), E217 (EMDB-29487) and phiTE (EMDB-45435). (TIF)

S14 Fig Structural comparisons of the baseplate in the extended P1 and other myophages. (A) Structural comparison of similar tube-like folds (green) among tail tube, tube initiator, tube-linking protein, and hub in P1. (B) Structural and sequence comparisons of the spike complex between P1 and T4. (C) Structural differences of the plug among myophages P1, E217 (PDB ID: 8EON), Pam3 (PDB ID: 7YFZ), and XM1 (PDB ID: 7KH1).
(TIFF)

S1 Table Refinement and model statistics of P1. (XLSX)
