# Supplementary figures and images for: Structure of defense against restriction proteins DarA and Hdf in phage P1 reveals a new molecular mechanism during phage assembly, infection and DNA ejection
Source: PLoS Pathog. 2026 Jan 16;22(1):e1013869. doi: 10.1371/journal.ppat.1013869 (PMC12810830; doi:10.1371/journal.ppat.1013869)

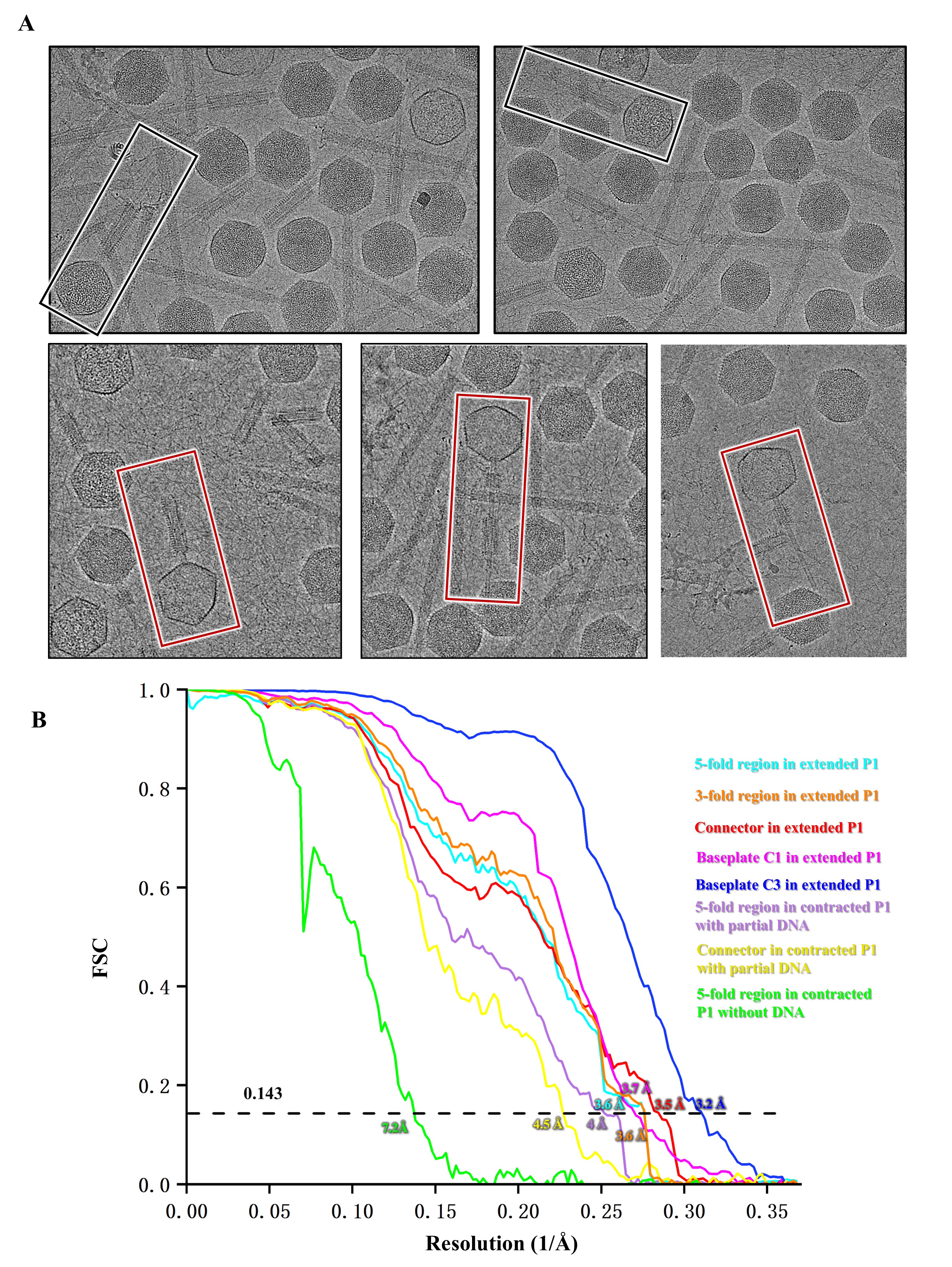

Supplement: S1 Fig — (A) Representative cryo-EM images of the extended and contracted P1. The contracted P1 with and without DNA was boxed black and red, respectively. (B) Estimated structural resolutions of the 5-fold region (3.6 Å), the 3-fold region (3.6 Å), the connector complex (3.5 Å), the baseplate (3.2 Å) imposing C3 symmetry and the baseplate (3.7 Å) imposing C1 symmetry in the extended P1, and the 5-fold region (4 Å), and the connector complex (4.5 Å) in the contracted P1 with partial DNA, as well as the 5-fold region (7.2 Å) in the contracted P1 without DNA. (TIFF) [file ppat.1013869.s001.tiff]

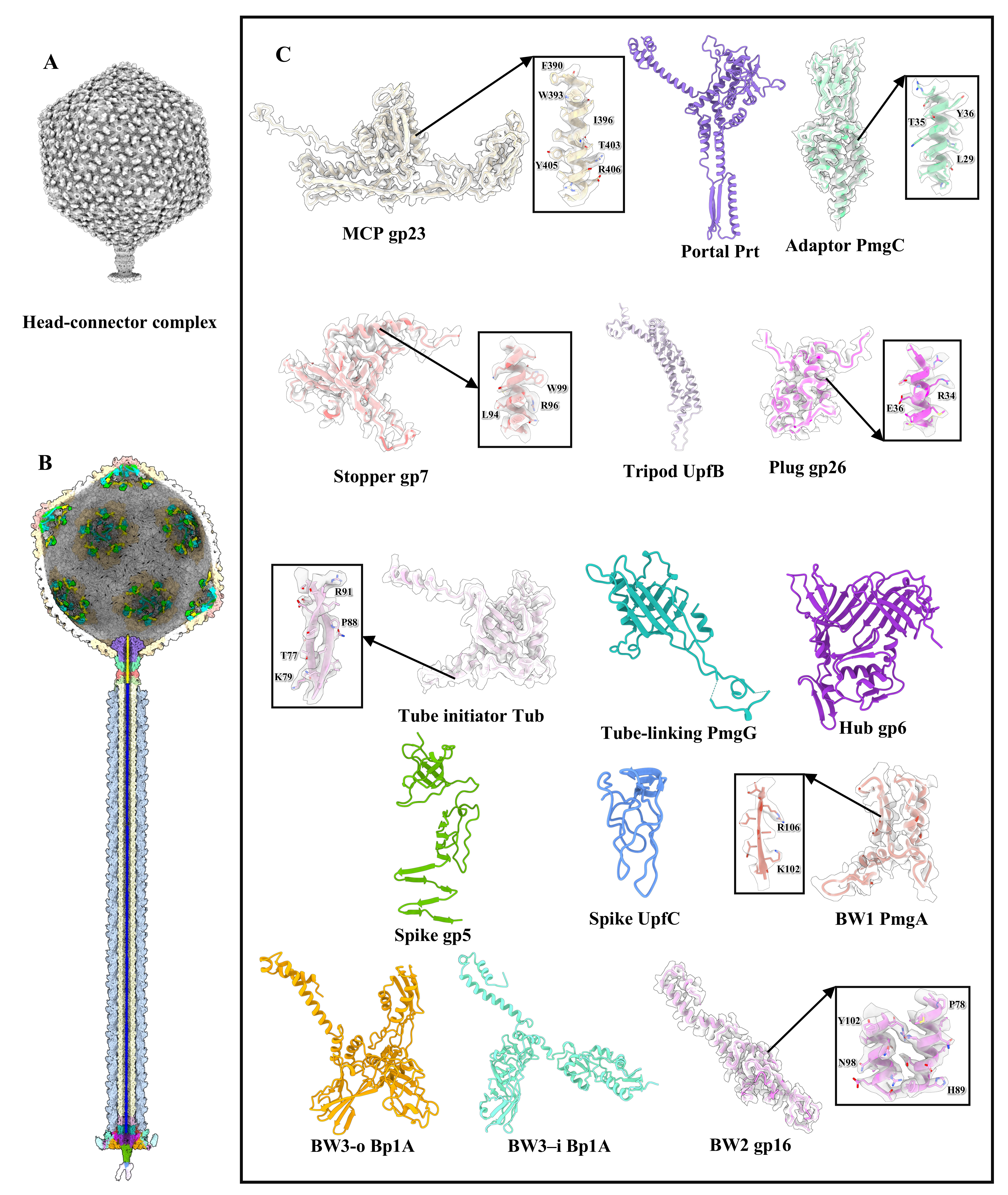

Supplement: S2 Fig — (A) Side views of the symmetry-mismatched structure of the head-connector complex in the extended P1. (B) Cut-open view of the intact structure of extended P1. DNA is manually removed to show the inner surface of head. Color codes are identical to that used in Fig 1B. (C) Ribbon models of almost all protein components from the head-tail and density maps (transparency) of partial protein components superimposed on their atomic models. All the atomic models were manually built, except for UpfB modelled by AlphaFold3. (TIFF) [file ppat.1013869.s002.tiff]

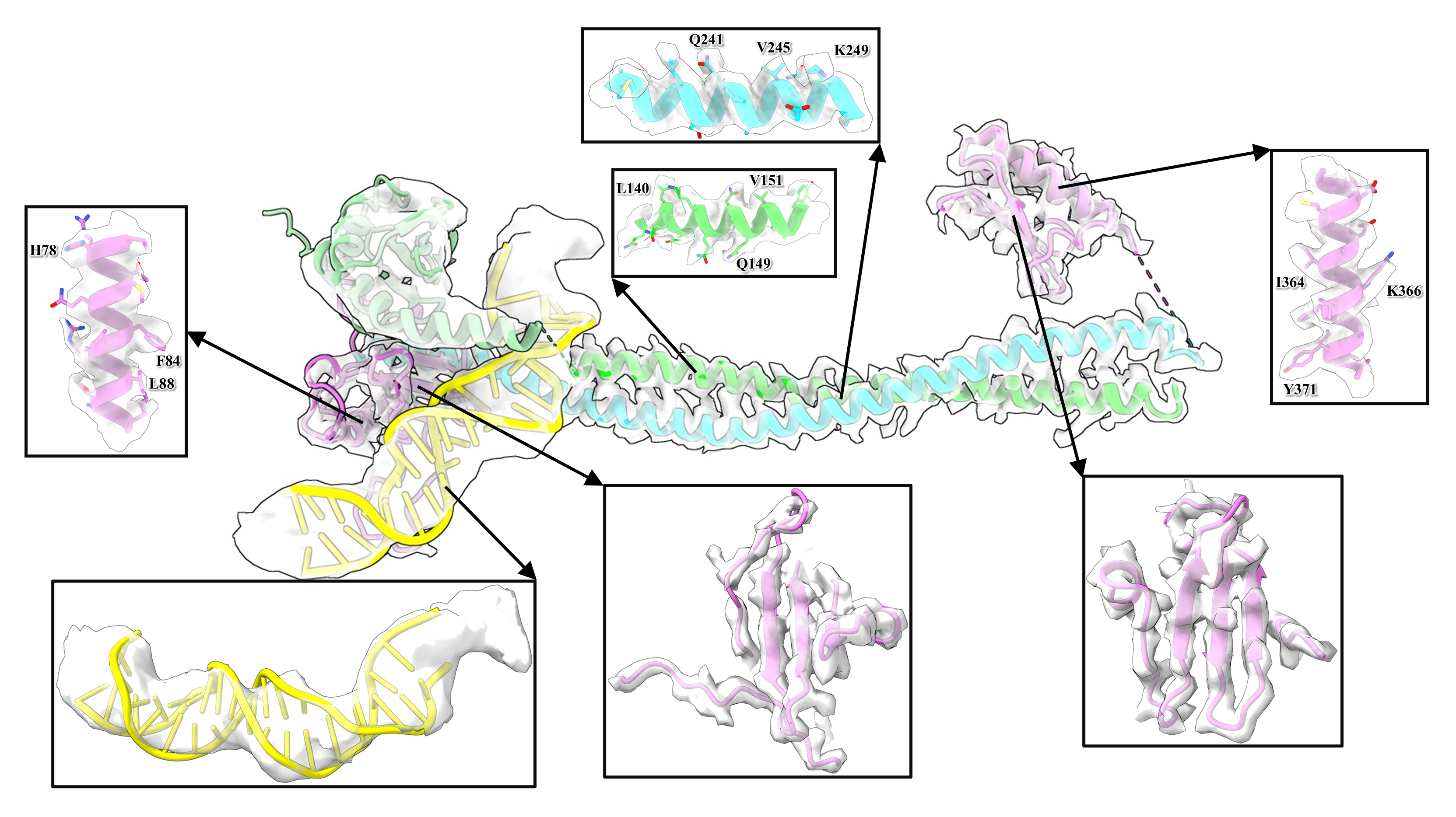

Supplement: S3 Fig — Density maps (transparency) of Dar proteins and DNA are superimposed on their atomic models. The color codes are identical to that in Fig 1D. (TIFF) [file ppat.1013869.s003.tiff]

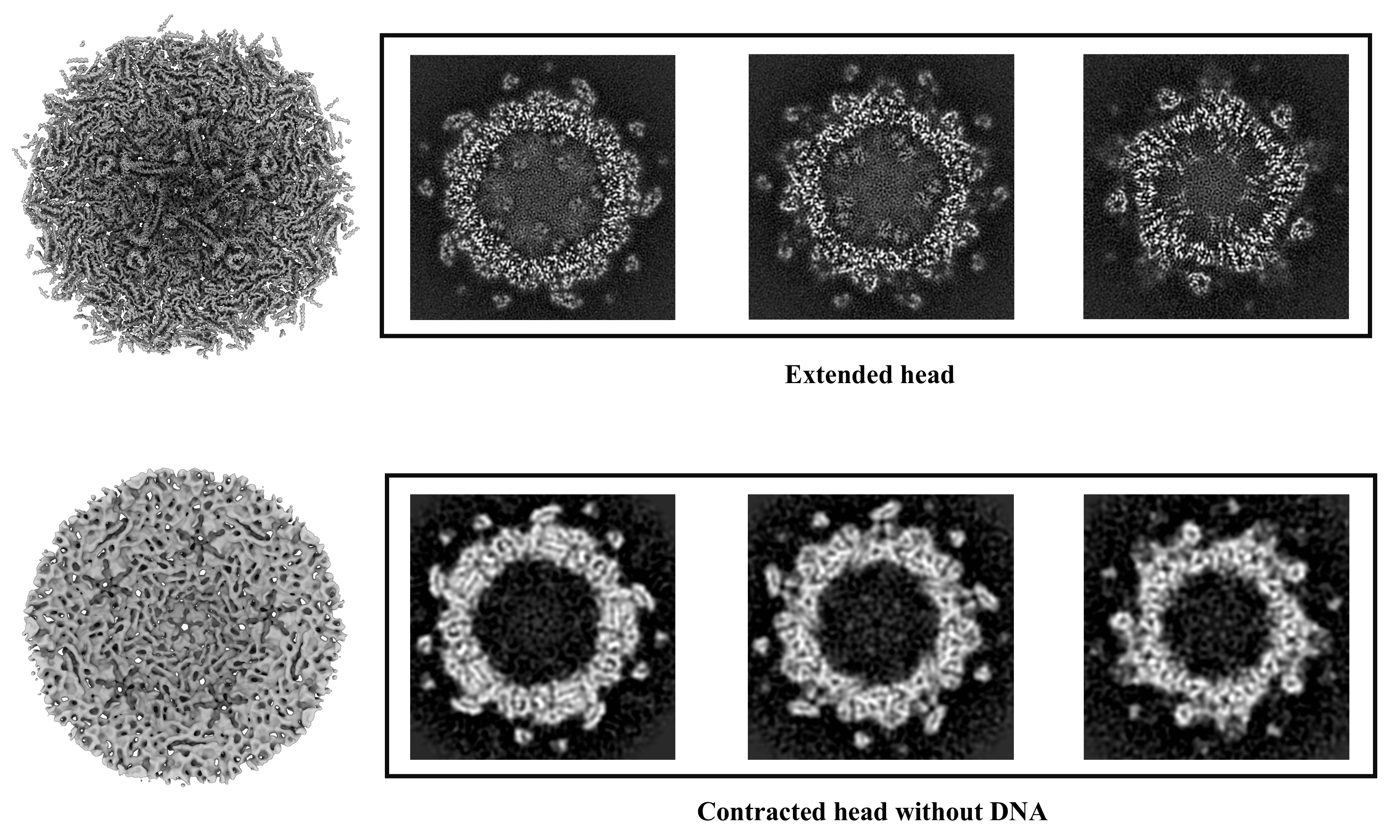

Supplement: S4 Fig — Left panel shows the bottom view of 5-fold vertex structure of the head. Right panel shows cross-sections at various thicknesses of the 5-fold vertex of the head. (TIFF) [file ppat.1013869.s004.tiff]

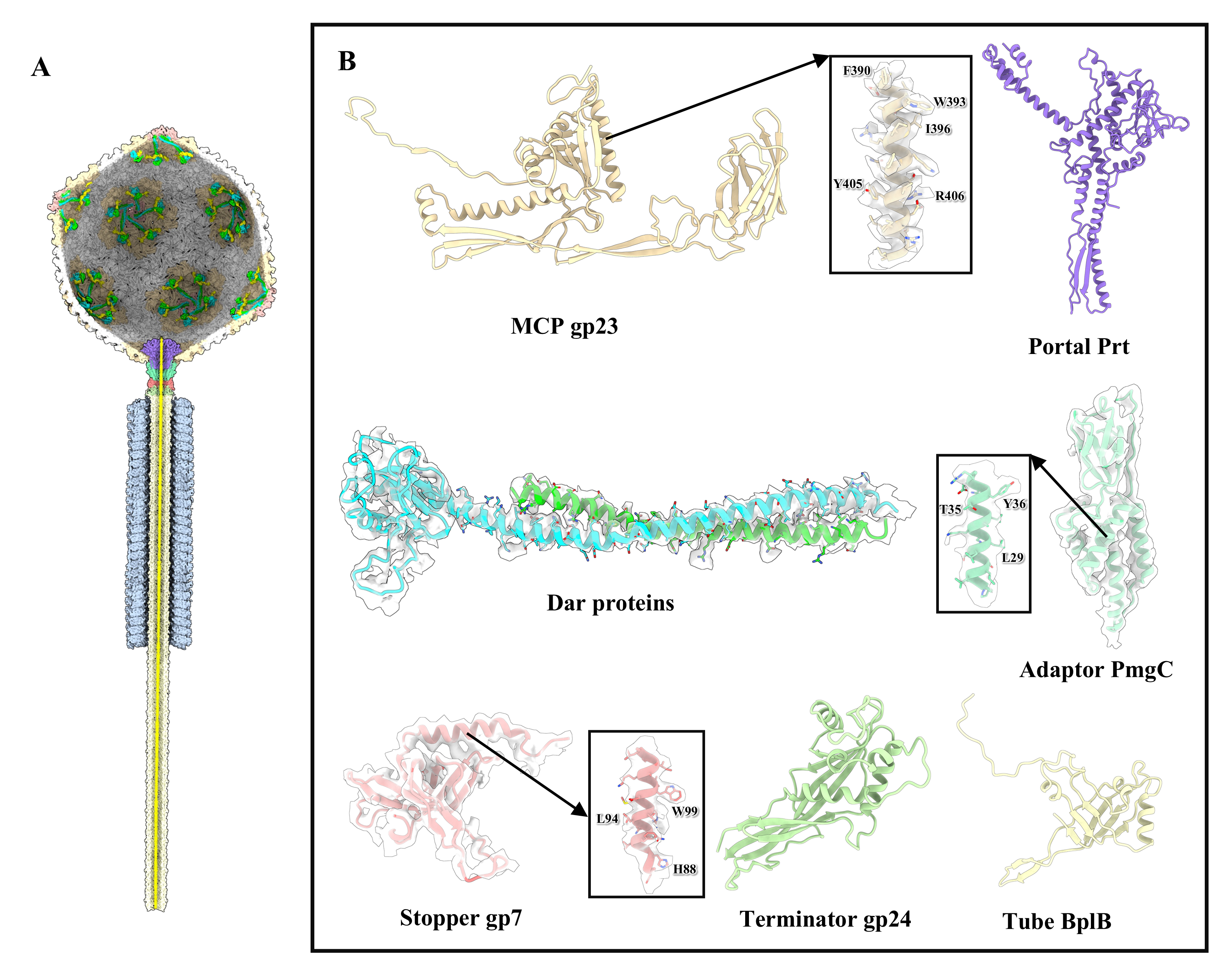

Supplement: S5 Fig — (A) Cut-open views of the head-tail in the contracted P1 with partial DNA. DNA is removed manually to show the inner surface of head. Color codes are identical to that used in Fig 1B. (B) Ribbon models of all protein components from the head-tube complex, and density maps (transparency) of partial protein components superimposed on its atomic models. (TIFF) [file ppat.1013869.s005.tiff]

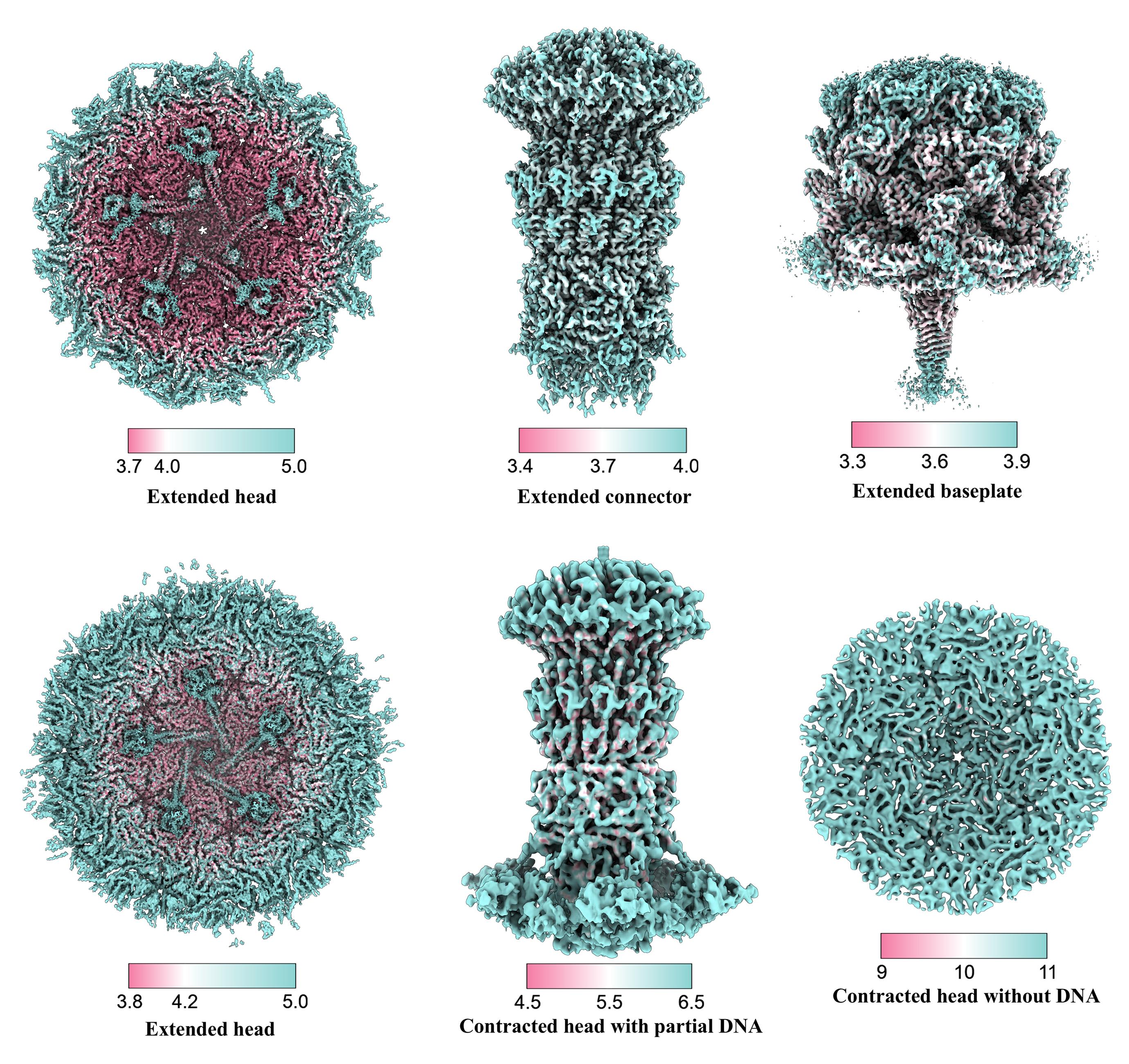

Supplement: S6 Fig — (TIF) [file ppat.1013869.s006.tif]

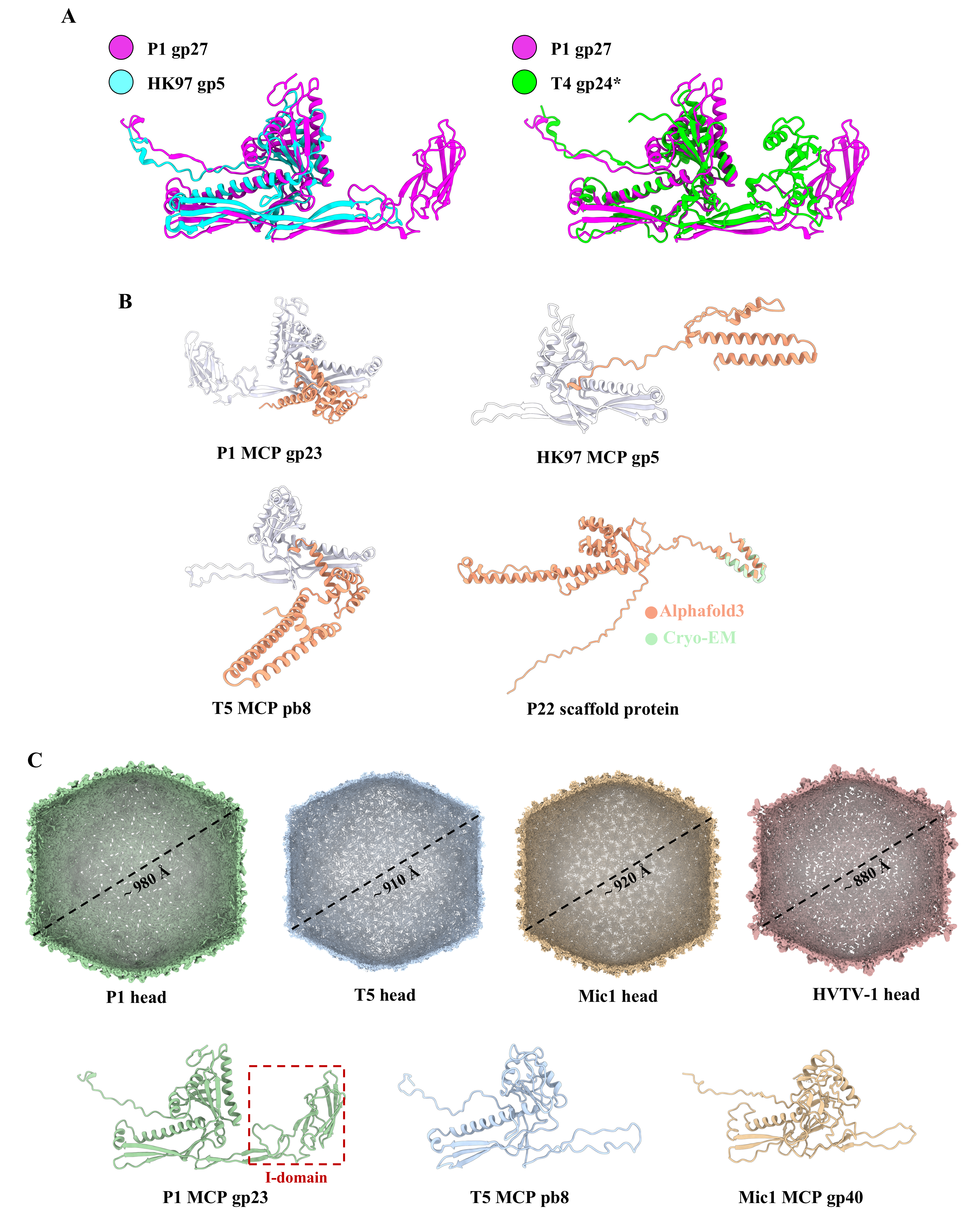

Supplement: S7 Fig — (A) Structural comparison between the P1 MCP and the HK97/T4 MCP (PDB ID: 1OHG/ 5VF3). (B) Structural comparisons of the scaffold domain or scaffold protein predicted by AlphaFold3 (copper colors) among P1, T5, HK97 and P22. (C) Top: Cut-open views of the density map of the icosahedral capsids, including P1, T5 (EMD-20125), Mic1 (EMD-9774) and HVTV-1 (EMD-2234). Bottom: Ribbon models of the MCP among P1, T5 (PDB ID: 6omc) and Mic1 (PDB ID: 6j3q). (TIFF) [file ppat.1013869.s007.tiff]

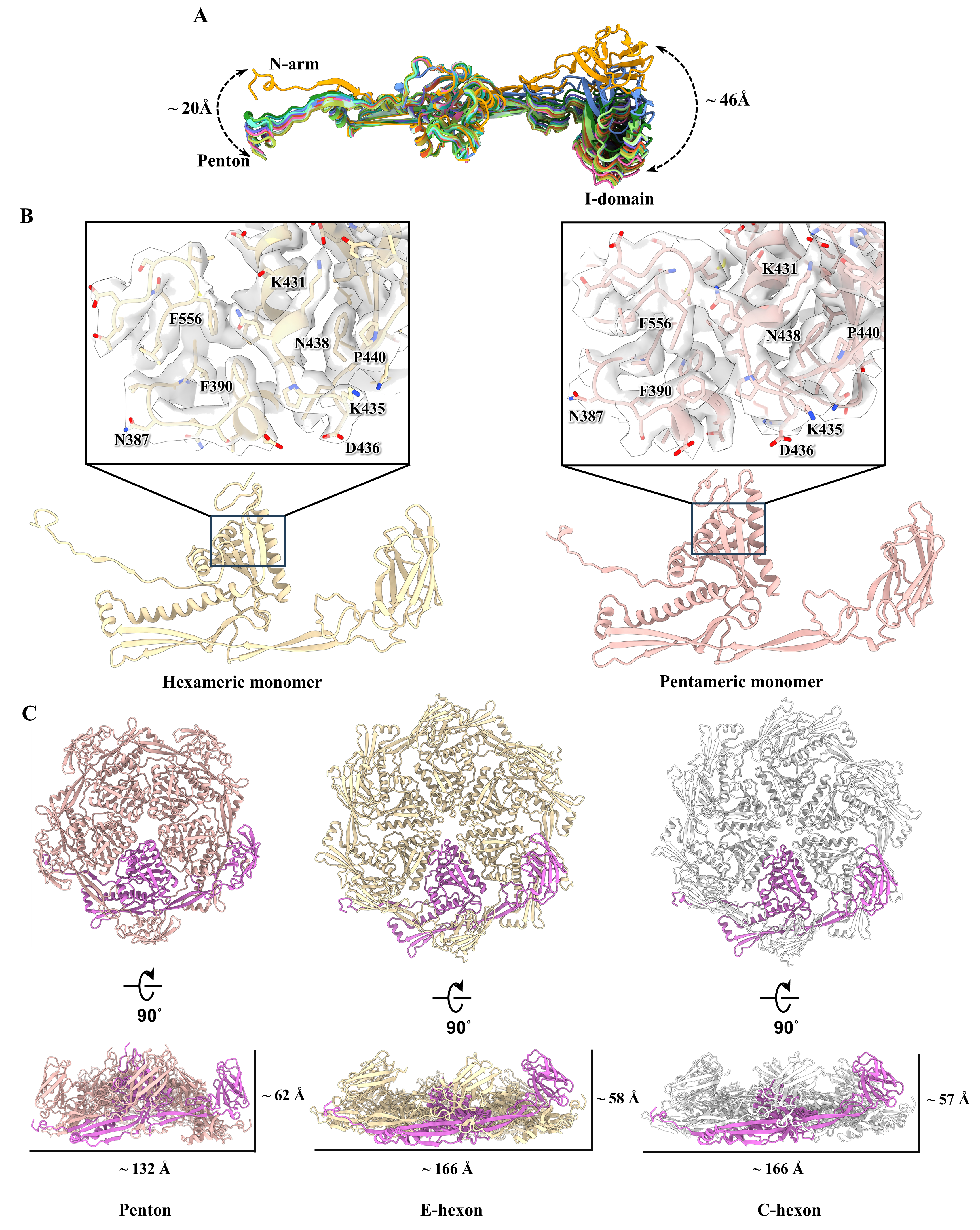

Supplement: S8 Fig — (A) Superimposition of the 13 MCPs, displayed in different colors. (B) Zoomed-in views of density maps (transparency) superimposed on atomic models of the hexameric and pentameric monomer (sticks). (C) Top and side views of ribbon models of the penton, E-hexon, and the C-hexon. Color codes are identical to that used in Fig 1B, except for a monomer of each capsomer colored in magenta. (TIFF) [file ppat.1013869.s008.tiff]

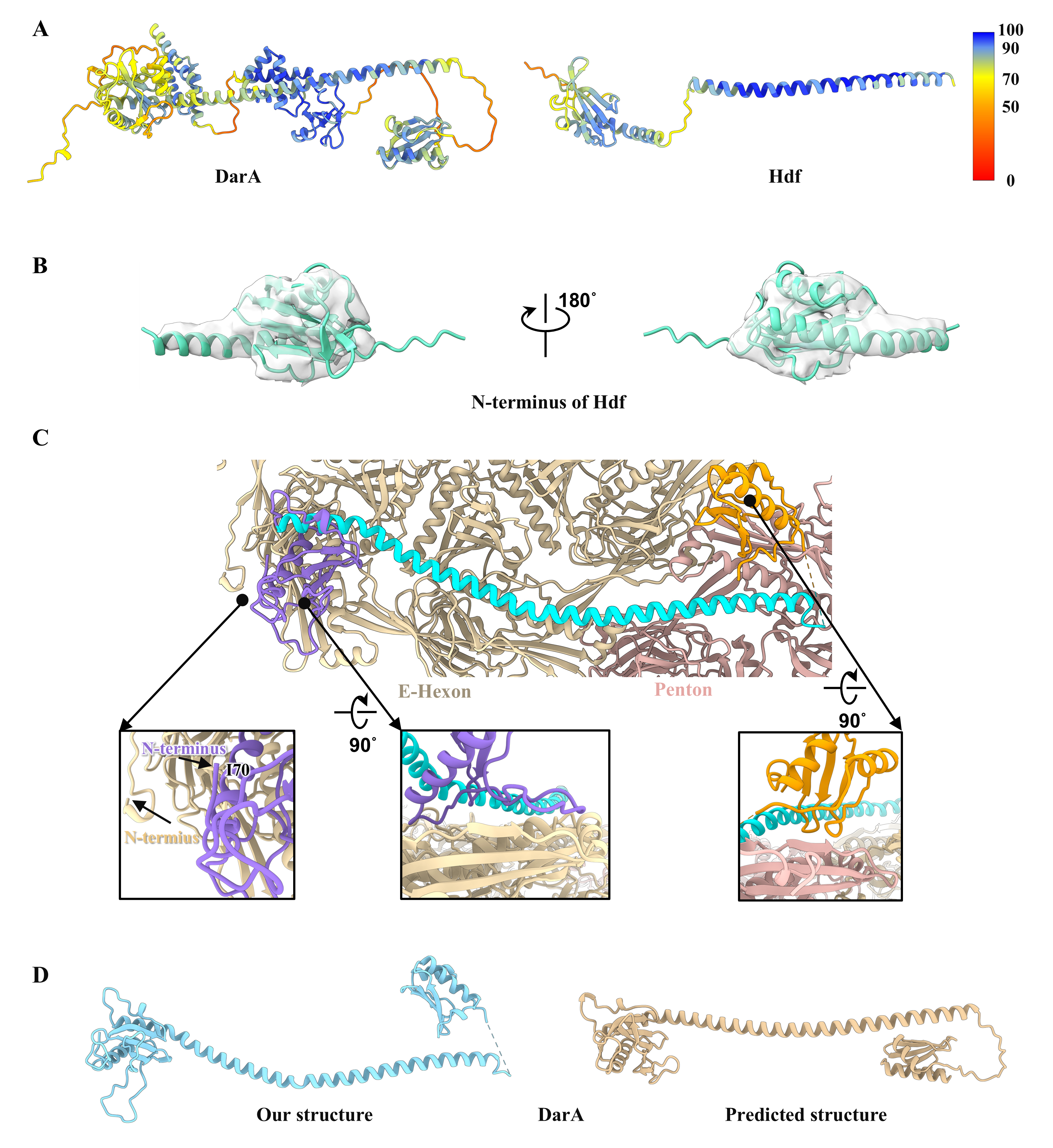

Supplement: S9 Fig — (A) Ribbon models of the DarA and Hdf predicted by AlphaFold3. The Predicted Local Distance Difference Test (pLDDT) is shown in the color bar. (B) Density map (transparency) of Hdf superimposed on its atomic model modelled by AlphaFold3. (C) Zoom-in views of the interactions between DarA and capsid. Color codes are identical to that used in Fig 3A. (D) Comparison of the N-terminus of DarA from the hexon binding domain to the penton binding domain between our structure and the predicted structure. (TIFF) [file ppat.1013869.s009.tiff]

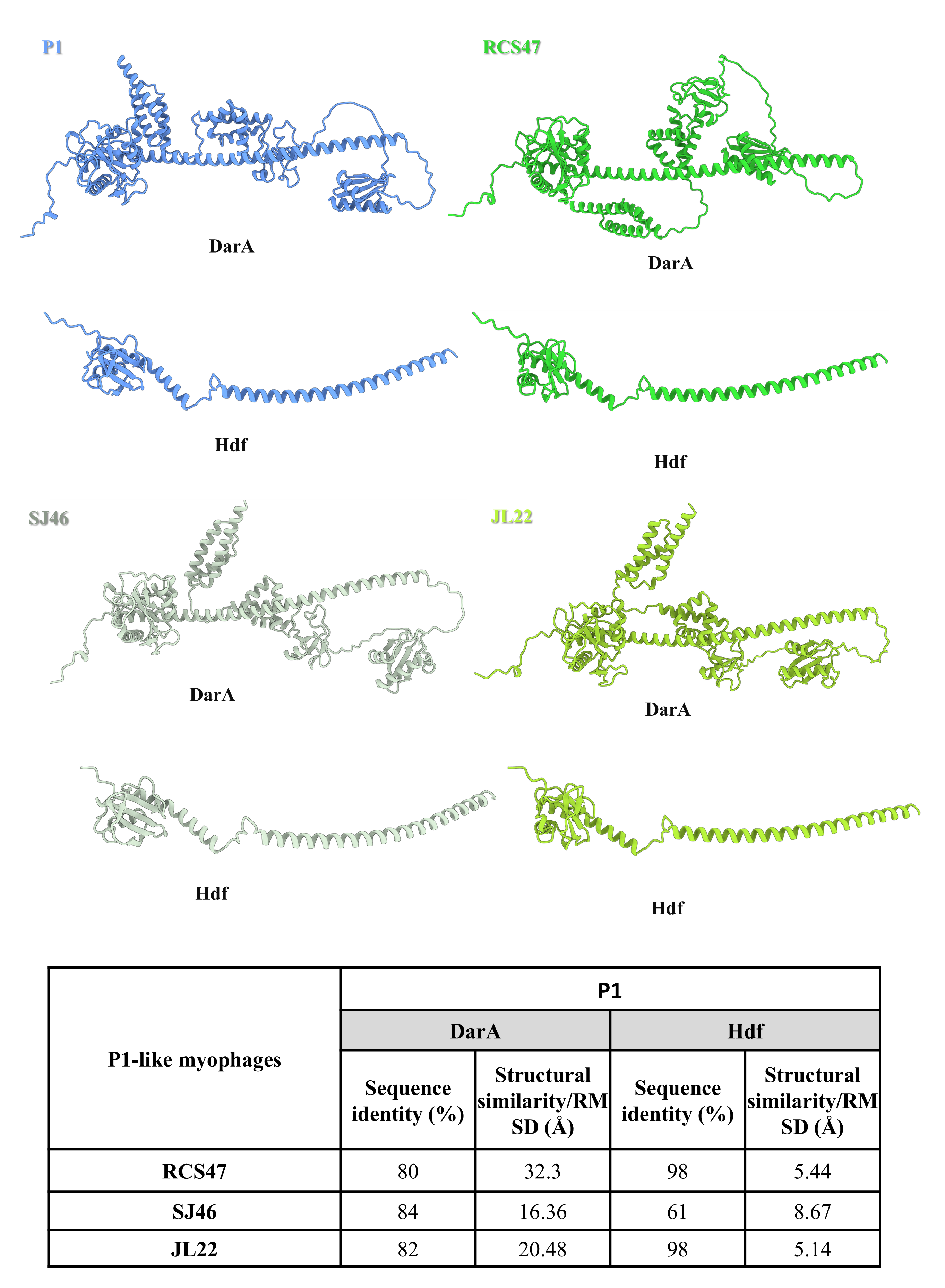

Supplement: S10 Fig — All structures of Hdf and DarA in P1-like phages are predicted by AlphaFold3. (TIFF) [file ppat.1013869.s010.tiff]

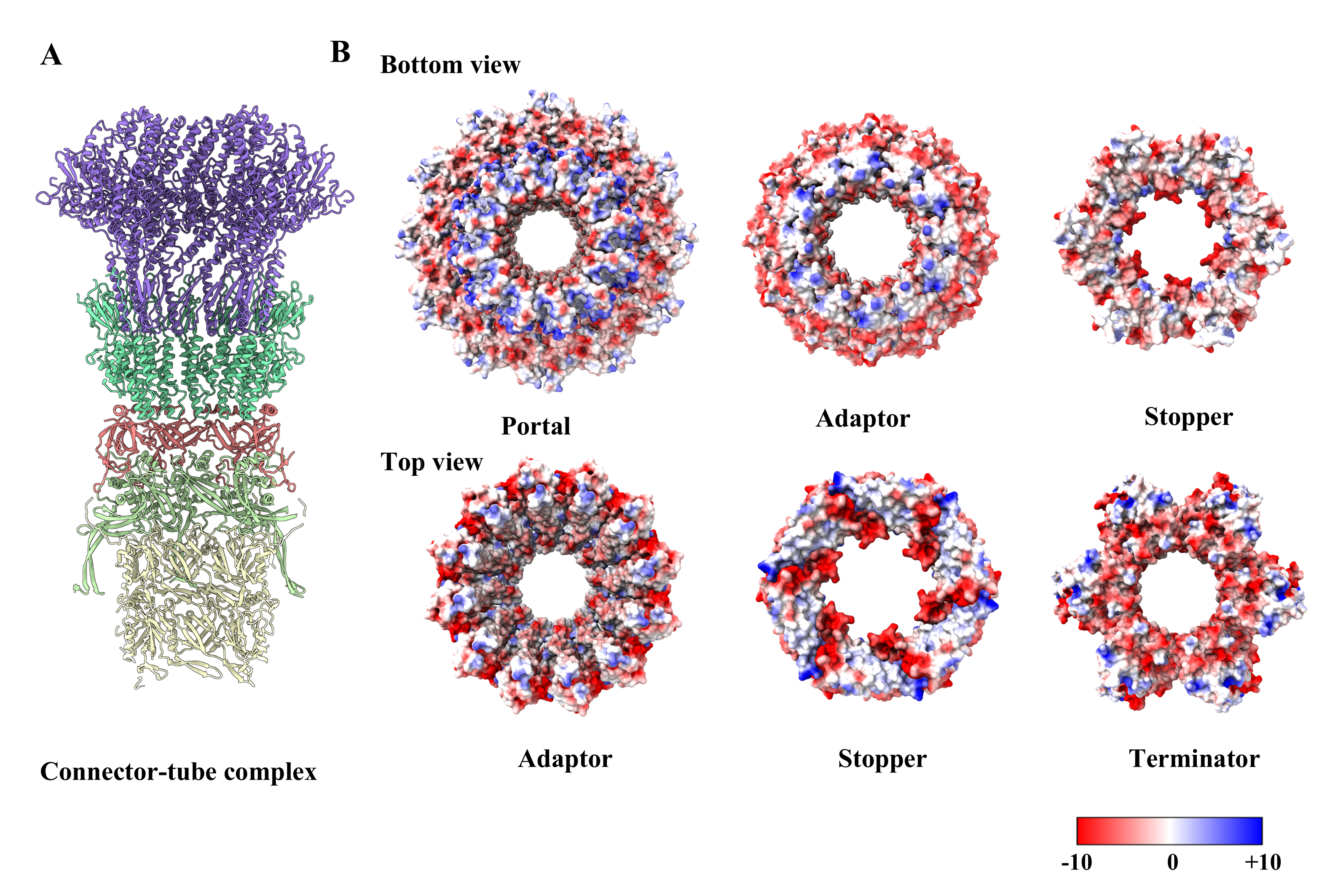

Supplement: S11 Fig — (A) Cut-open view of ribbon models of the connector-tube complex (tube PDB ID: 8jan). Color codes are identical to that used in Fig 1B. (B) Electrostatic potential surfaces between two adjacent protein components among the connector complex and tail terminator. The top columns are oriented toward the tail, whereas the bottom columns are oriented toward the head. The electrostatic potential scale is shown in the color bar. (TIFF) [file ppat.1013869.s011.tiff]

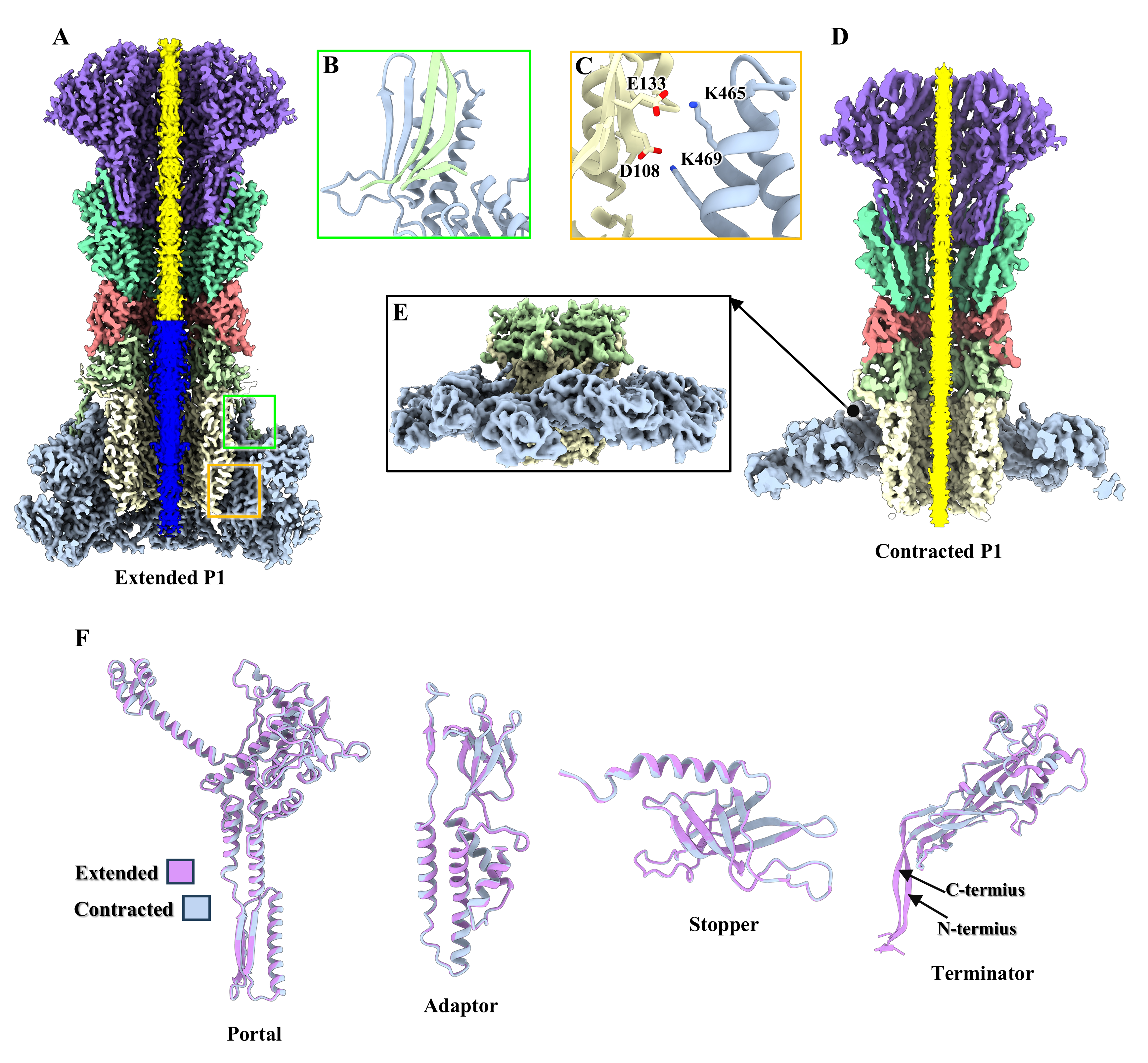

Supplement: S12 Fig — (A, D) Cut-open views of the density map from the connector complex to the tail in the extended (A) and contracted (D) P1. Color codes are identical to that used in Fig 1B. (B, C) Zoomed-in views of the box regions in panel A to show the interactions among the tail terminator-sheath (B) and tail tube-sheath (C) of the extended P1. (E) Zoomed-in view of the interactions between the tail terminator, tail tube and tail sheath of the contracted P1. (F) Structural comparisons of the portal, adaptor, stopper and terminator in the extended and contracted P1. (TIFF) [file ppat.1013869.s012.tiff]

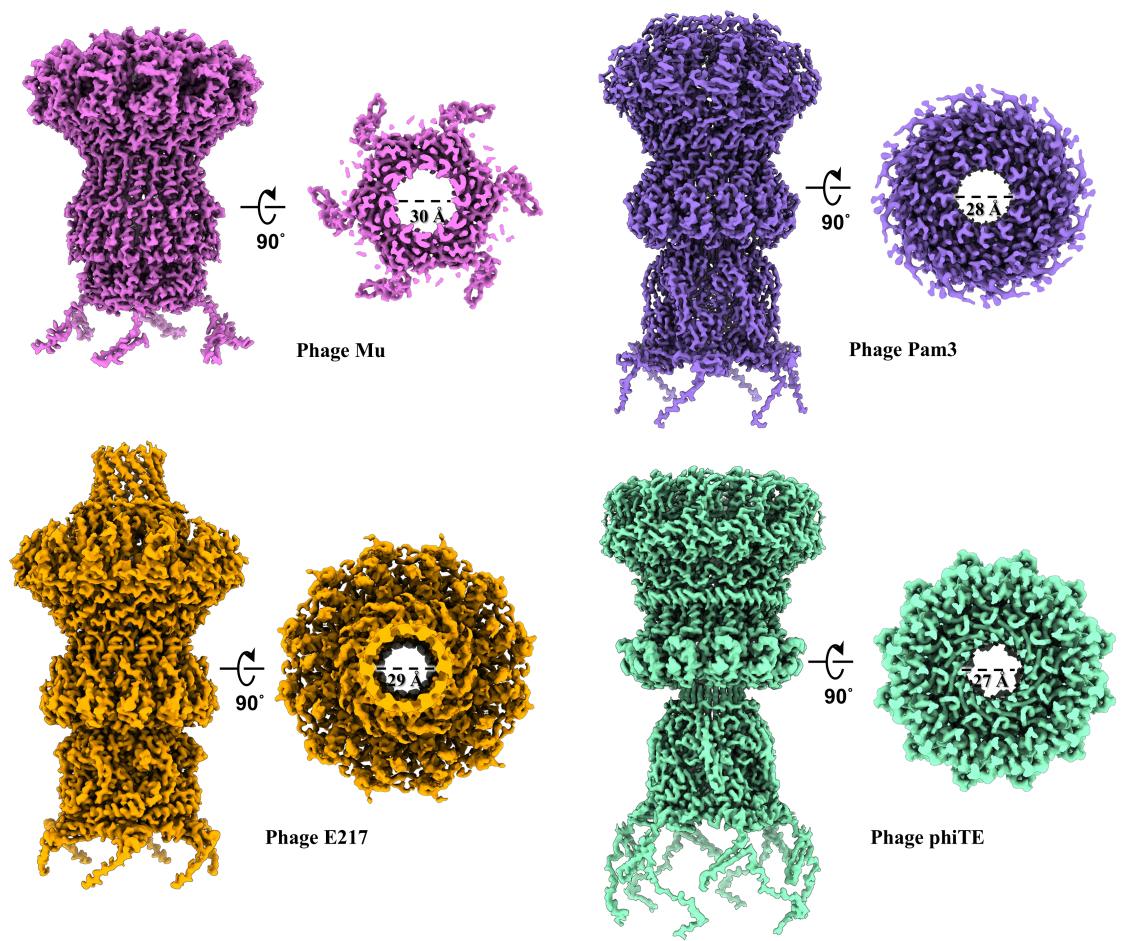

Supplement: S13 Fig — (TIF) [file ppat.1013869.s013.tif]

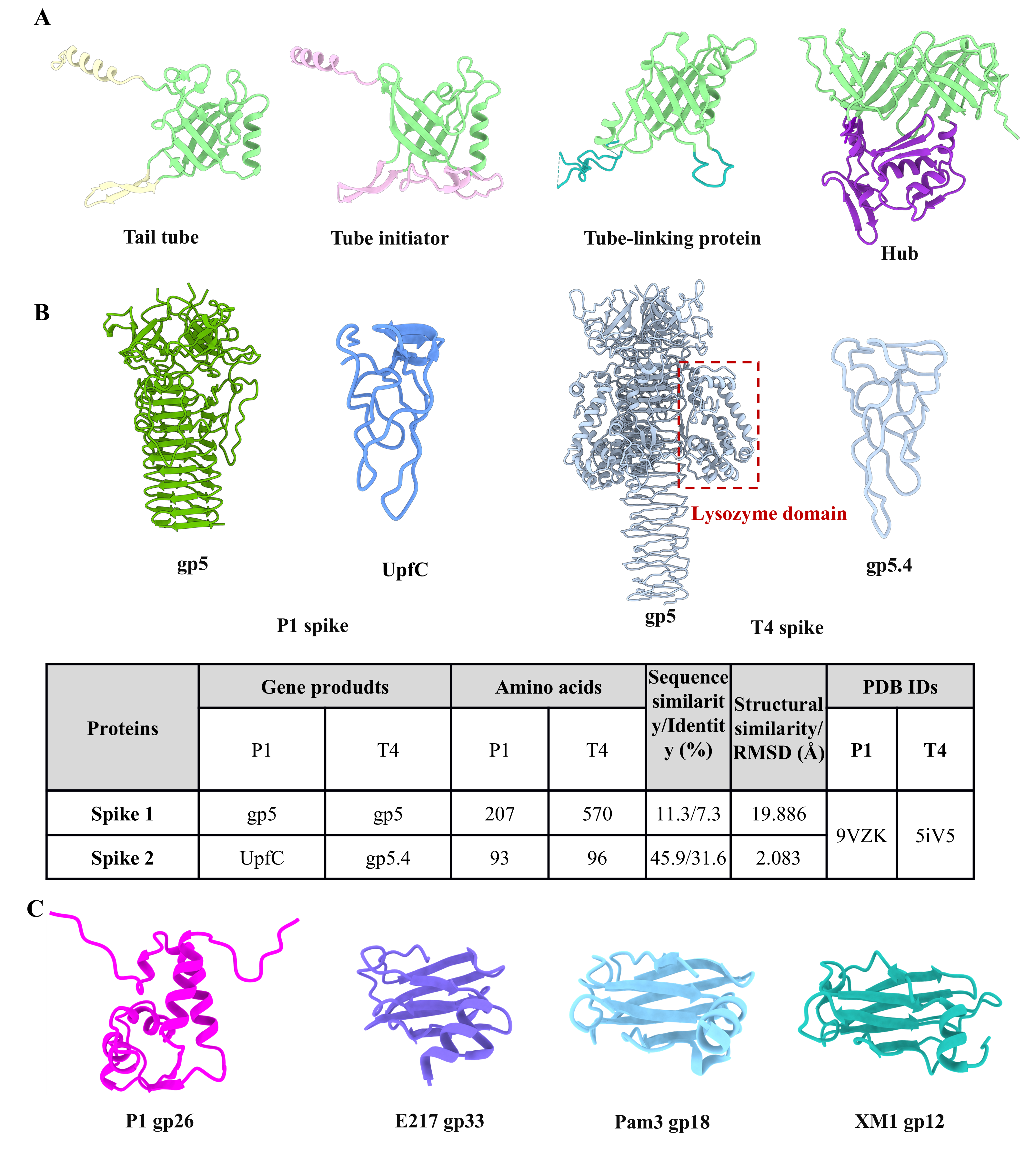

Supplement: S14 Fig — (A) Structural comparison of similar tube-like folds (green) among tail tube, tube initiator, tube-linking protein, and hub in P1. (B) Structural and sequence comparisons of the spike complex between P1 and T4. (C) Structural differences of the plug among myophages P1, E217 (PDB ID: 8EON), Pam3 (PDB ID: 7YFZ), and XM1 (PDB ID: 7KH1). (TIFF) [file ppat.1013869.s014.tiff]
